# Probabilistic deconvolution of PET images using informed priors

**DOI:** 10.3389/fnume.2022.1028928

**Published:** 2023-01-12

**Authors:** Thomas Mejer Hansen, Klaus Mosegaard, Søren Holm, Flemming Littrup Andersen, Barbara Malene Fischer, Adam Espe Hansen

**Affiliations:** ^1^ Department of Geoscience, Aarhus University, Aarhus, Denmark; ^2^ Physics of Ice, Climate and Earth, Niels Bohr Institute, University of Copenhagen, Copenhagen, Denmark; ^3^ Department of Clinical Physiology, Nuclear Medicine and PET, Rigshospitalet, University of Copenhagen, Copenhagen, Denmark; ^4^ Department of Clinical Medicine, University of Copenhagen, Copenhagen, Denmark; ^5^ Cancer Imaging, School of Biomedical Engineering and Imaging Sciences, King's College London, London, United Kingdom; ^6^ Department of Radiology, Rigshospitalet, University of Copenhagen, Copenhagen, Denmark

**Keywords:** PET, probabalistic approach, quantitative, statistical, deconvolution, positron emission tomography

## Abstract

**Purpose:**

We present a probabilistic approach to medical image analysis that requires, and makes use of, explicit prior information provided by a medical expert. Depending on the choice of prior model the method can be used for image enhancement, analysis, and segmentation.

**Methods:**

The methodology is based on a probabilistic approach to medical image analysis, that allows integration of 1) arbitrarily complex prior information (for which realizations can be generated), 2) information about a convolution operator of the imaging system, and 3) information about the noise in the reconstructed image into a posterior probability density. The method was demonstrated on positron emission tomography (PET) images obtained from a phantom and a patient with lung cancer. The likelihood model (multivariate log-normal) and the convolution operator were derived from phantom data. Two examples of prior information were used to show the potential of the method. The extended Metropolis-Hastings algorithm, a Markov chain Monte Carlo method, was used to generate realizations of the posterior distribution of the tracer activity concentration.

**Results:**

A set of realizations from the posterior was used as the base of a quantitative PET image analysis. The mean and variance of activity concentrations were computed, as well as the probability of high tracer uptake and statistics on the size and activity concentration of high uptake regions. For both phantom and in vivo images, the estimated images of mean activity concentrations appeared to have reduced noise levels, and a sharper outline of high activity regions, as compared to the original PET. The estimated variance of activity concentrations was high at the edges of high activity regions.

**Conclusions:**

The methodology provides a probabilistic approach for medical image analysis that explicitly takes into account medical expert knowledge as prior information. The presented first results indicate the potential of the method to improve the detection of small lesions. The methodology allows for a probabilistic measure of the size and activity level of high uptake regions, with possible long-term perspectives for early detection of cancer, as well as treatment, planning, and follow-up.

## 1. Introduction

The purpose of medical imaging is to provide images of pertinent features and properties of the interior of the body, that can be used by medical experts to, for example, diagnose diseases, design a course of treatment, and monitor the effects of treatment.

Imaging methods such as computed tomography (CT) and magnetic resonance imaging (MRI) are widely used for anatomical and structural imaging but have also physiological and functional applications. Positron emission tomography (PET) and single-photon emission computed tomography (SPECT) have less spatial resolution than CT and MRI but have unique capabilities and sensitivity for metabolic and molecular imaging (1, 2).

PET imaging is a medical imaging method in which a radioactive tracer is injected into the body. The radioactive positron emitter gives rise to pairs of collinear photons being emitted from the location of the tracer in the human body. The photons are registered by a detector ring and from this information, a 3D volume of the tracer distribution can be reconstructed. A tracer such as the 2-[^18^F]-fluoro-2-deoxy-D-glucose (FDG) tracer, is trapped intracellularly and has a higher uptake in cancerous and inflammatory than in healthy tissues due to the generally high energy consumption of cancer and inflammatory cells. Therefore, FDG-PET is a widely used clinical tool to locate and characterize cancer (3).

The goal of PET tomography is to estimate the *in vivo* distribution of tracer uptake in the body. This can be done using for example variants of filtered backprojection (FBP) (4), or iterative methods such as the maximum likelihood expectation-maximization (MLEM) method (5), and using ordered subsets expectation-maximization (OSEM) (6). It can also be done using maximum a posteriori methods (MAP) (7). The resolution and noise in the output image depend on the reconstruction method, the number of iterations and number of used subsets (using OSEM), and the use of a system matrix and point spread function (8, 9). See for example (10) for an overview of PET image reconstruction methods. In any case, we refer to the outcome of any such (usually iterative) reconstruction algorithm as the “reconstructed PET image.”

Analysis of reconstructed PET images is difficult due to 1) noise in the PET image and 2) the relatively low resolution of PET images as compared to, for example, images obtained using MRI or CT. The low-resolution results from a combination of the physical properties of the detector system and the positron range of the PET tracer. In practice, this means that the activity of adjacent regions will be mixed in the PET image. The resulting Partial Volume Effect (PVE) especially affects tracer uptake in small tumors (11). The Point Spread Function (PSF) refers to the PET image one would obtain by scanning a point source and is a way to quantify the system resolution and the smoothing inherent in the formation of the image (12). The PSF depends on the scanner geometry, properties of the crystals in the detector, the radionuclide used, and the use of a reconstruction algorithm typically without any resolution modeling (13). The PSF can be estimated both experimentally, and theoretically (14–17).

Several methods have been developed to address the smoothing, noise, and limited spatial resolution related to reconstructed PET images. Some of these are referred to as Partial Volume Correction (PVC) or PSF methods (11, 12, 18). Both reconstruction and post-reconstruction techniques can incorporate PSF deconvolution and/or other image types. One class of methods is applied during reconstruction, for example by including the PSF directly in the system matrix (15, 19–22). Another class of methods is applied as post-reconstruction techniques, sometimes referred to as image restoration (11, 12, 23–26).

In more general terms, deconvolution of a reconstructed PET image can be used to infer information about the *in vivo* distribution of uptake (27, 28). But, straightforward deconvolution tends to amplify the noise of the reconstructed PET image and introduce “ringing” Gibbs artifacts in the PET image. Gibbs artifacts can be removed by applying a smoothing filter, which leads to a drop in resolution. The Gibbs artifacts stem from the fact that any PET scanning system is insensitive to higher frequency variations of the distribution of uptake, due to the physical construction of the scanner (17). One can choose to de-noise the PET image (29, 30) or make use of *a priori* information to introduce some of the higher frequencies not detected by the PET scanner, as anatomical priors from MRI or CT (23, 24). See e.g. (12) for a review of PVC-based methods. Cabello and Ziegler (31) provides a review of current imaging methods for combined PET/MR data.

All reconstruction and restoration methods are based on either a deterministic or probabilistic approach. Using deterministic methods, such as FBP, the goal is to find one PET image, that typically minimizes some objective function. Subsequently a local uncertainty estimate can be computed (5, 14, 32, 33). Probabilistic/Bayesian methods allow (and require) the specification of prior information (7, 34–37), and the solution is a probability distribution, the posterior distribution, that allows full uncertainty analysis. However, in practice, this can be intractable to compute, and instead, two different approaches can be taken to infer information about the posterior distribution: optimization and sampling.

The most applied probabilistic method in medical imaging is the use of non-linear optimization methods (e.g. MLEM, OSEM) to locate a PET image, for example the one with maximum likelihood or maximum a posteriori probability, i.e. the MAP image (5–7, 38–41), sometimes based on regularizing edge-preserving prior models (42, 43). In some cases, the uncertainty of the related PET image obtained using optimization can also be estimated using analytical approximations (32).

A less widely used probabilistic approach in medical imaging is the sampling approach (44–46). Here the goal is to generate a sample of the posterior distribution, i.e. a collection of PET images that occur with a frequency proportional to the posterior distribution. Given a large enough sample any statistical property of the posterior distribution can be estimated, and the method allows full uncertainty analysis. Sampling methods are typically computationally demanding, and also, it may be nontrivial to quantify prior information such that it can be used with sampling methods. Filipović et al. (46) propose such a Bayesian sampling method for PET reconstruction using prior information from MR data, along with a prior based on the distance-dependent Chinese Restaurant Process (ddCRP) (47).

The purpose of this paper is to introduce a probabilistic method for the analysis of a reconstructed PET image, based on using available information, such as about the effective PSF, a statistical model describing the noise in the reconstructed PET image, and an explicit choice of a statistical model describing the prior information, that should preferably be chosen by a medical expert.

Specifically, the methodology allows relatively easy use of a large collection of prior model types derived from geostatistical simulation. These vary from simple multivariate Gaussian-based models to multiple-point statistical models that allow quantifying complex spatial patterns and features (48, 49). We hypothesize that the method can both increase resolution and decrease noise simultaneously, without producing artifacts such as Gibbs ringing.

The output of the method is not a single PET image (such as the MAP image), but instead a collection of PET images of the tracer activity concentration, that represent a sample of the posterior. Each of these PET images will by construction be consistent with available information. We aim to demonstrate how such a sample from the posterior distribution can be used as a quantitative tool for reconstructed PET image analysis, to for example assess, with uncertainty, the size and activity concentration of regions of interest, such as high activity regions indicative of cancer lesions.

In section 2 we lay out the theory, and propose a methodology for probabilistic PET image analysis in the image domain (i.e. based on a reconstructed PET image). We demonstrate the method for a specific choice of PET scanner (Siemens Biograph mMR) and PET reconstruction method (OP-OSEM). Two data sets are considered, a phantom and an in vivo case, and described in section 3. An example of how to quantify the noise model (3.2.2), PSF (3.2.1) is provided, and two examples of prior information (3.3) are used here to show the potential of the method. Results of applying the methodology are given in sections 4 and 5.

## 2. Theory and method

In the following, let $\Phi =[\phi_{1},\phi_{2},\ldots ,\phi_{M}]$ represent M model parameters that define the in situ PET activity concentration within M voxels. A pixel will refer to a voxel in the x–y plane. A specific set of model parameters represents a point in a high-dimensional model parameters space. Each point (set of model parameters) in the model parameter space refers to a specific PET image (in 2D or 3D).

A PET scanner measures the counts of pairs of photons, at different locations, caused by positron emission decay of the radionuclide (injected into a patient) whos activity is Φ. The PET reconstruction problem, is then the inverse problem, of inferring information about Φ given the observed data.

In the following $\Phi_{\mathrm{PET}}=[\phi_{{\mathrm{PET}}_{1}},\phi_{{\mathrm{PET}}_{2}},\ldots ,\phi_{{\mathrm{PET}}_{N}}]$, consisting of N pixel values, represents a noise-free reconstructed PET image obtained using a specific reconstruction method such as for example FBP, MLEM, or OSEM (5, 6, 15, 19–22, 50). The relation g (analytical and/or numerical) between the model parameters Φ and the noise free PET image $\Phi_{\mathrm{PET}}$ is given by

(1) $$\Phi_{\mathrm{PET}}=g(\Phi ).$$

g consists of a physical mapping of the model parameters Φ into photon counts, followed by an algorithmic reconstruction. In the remainder of the text we make use of a linear smoothing operator, G, such that relation, Eq. 1, reduces to

(2) $$\Phi_{\mathrm{PET}}=G\Phi ,$$

and refer to a convolution problem. Note that if a non-linear forward operator and/or algorithm exist it can trivially be used with the methodology presented below.

Let $\Phi_{\mathrm{PET}}^{\mathrm{obs}}$ represent an actual observed reconstructed PET image (as obtained from PET reconstruction of scanning a target) that will never be the same as the noise free forward response $\Phi_{\mathrm{PET}}$, due to noise. In a probabilistic formulation the uncertainty in the reconstructed PET image is defined by a probability density $\rho_{\Phi }(\Phi_{\mathrm{PET}})$, expressing the distribution of the devations between the noise free PET image $\Phi_{\mathrm{PET}}$ and $\Phi_{\mathrm{PET}}^{\mathrm{obs}}$, which will be referred to as the noise distribution, following (34).

The properties of the noise in a reconstructed PET image depends on multiple factors such as machine type, injection dose, and the type of reconstruction method used (8, 32, 51–56). In general, using MLEM and OSEM leads to correlated noise where the variance is linked to the local mean. The higher the mean activity, the higher the variance. Also, the longer iterative MLEM and OSEM algorithms are run, the higher the variance of the noise becomes (8, 51). Therefore, in practice, such optimization algorithms are not run to convergence, but rely on early stopping, using a fixed limited number of iterations. Reconstruction of large homogenous phantoms leads to non-stationary correlated noise using FBP, but stationary noise using OSEM (53). The single pixel noise properties is represented well by normal distribution using FBP, (55). Using EM leads to a skewed single pixel noise distribution which can be represented by both a multivariate log-normal distribution using high photon count (low noise) (32, 54), and a gamma distribution using low photon count (higher noise) (56).

If the noise is considered multivariate Gaussian, with mean $\Phi_{\mathrm{PET}}^{\mathrm{obs}}$ and data covariance $C_{D}$, as when using FBP, then $\rho_{\Phi }(\Phi_{\mathrm{PET}})$ is given by

(3) $$\rho_{\Phi }(\Phi_{\mathrm{PET}})=\frac{1}{(2\pi {)}^{N/2}|C_{D}{|}^{1/2}}\exp(-0.5\;(\Phi_{\mathrm{PET}}-\Phi_{\mathrm{PET}}^{\mathrm{obs}}{)}^{T}C_{D}^{-1}(\Phi_{\mathrm{PET}}-\Phi_{\mathrm{PET}}^{\mathrm{obs}})),$$

Likewise, if the noise is multivariate log-normal, with mean $\log(\Phi_{\mathrm{PET}}^{\mathrm{obs}})$ and data covariance $C_{t}$ in log image space, such as when using OSEM, then $\rho_{\Phi }(\Phi_{\mathrm{PET}})$ is given by Kleiber and Kotz (57)

(4) $$\begin{array}{r} \rho_{\Phi }(\Phi_{\mathrm{PET}})=\frac{1}{(2\pi {)}^{N/2}|C_{t}{|}^{1/2}\prod_{i}^{N}\Phi_{{\mathrm{PET}}_{i}}}\\ \times \exp(-0.5(\log(\Phi_{\mathrm{PET}})\\ -\log(\Phi_{\mathrm{PET}}^{\mathrm{obs}}){)}^{T}C_{t}^{-1}(\log(\Phi_{\mathrm{PET}})\\ -\log(\Phi_{\mathrm{PET}}^{\mathrm{obs}}))). \end{array}$$

The same number of pixels/model parameters (and hence pixel size) can be used for the reconstructed PET image $\Phi_{\mathrm{PET}}$ and the model parameters describing the underlying activity distribution Φ, such that N=M, but this need not be the case, as will be demonstrated. The chosen pixel size of Φ provides a lower limit of the small scale variability that can be resolved. It should therefore be chosen small enough to be able to represent the resolution provided by whatever inversion/deconvolution used (58).

Here we consider the post-reconstruction problem of inferring information about the in situ activity concentration, Φ, related to an already reconstructed noisy PET image, $\Phi_{\mathrm{PET}}^{\mathrm{obs}}$. Equation 2 represent a convolution, and hence inferring information about Φ can be posed as a problem of deconvolution with noisy data. This deconvolution problem has been widely investigated as an optimization problem (12, 15, 19–22, 27, 28, 38, 59).

Below we present the problem of reconstructed PET image deconvolution in a probabilistic formulation following (60). The fundamental differences to most deconvolution methods are that 1) the methodology allows the incorporation of, in principle, arbitrarily complex prior information, and 2) the solution is not one single optimal image, but instead a collection of PET images from the posterior probability distribution representing the combined information and uncertainty of Φ. This can be used as a base for a quantitative approach to reconstructed PET image analysis, which will be demonstrated later.

### 2.1. Probabilistic deconvolution of a reconstructed PET image

Tarantola and Valette (34) present inverse problem theory through the concept of “conjunction of information” which is a probabilistic framework for integration of information. The solution is not one single optimal image, but instead, a *posterior* probability distribution that represents the combined information. Such a probability distribution represents, in principle, infinitely many images, and the uncertainty and resolution can be analyzed by analyzing such a set of realized images.

The a posteriori probability distribution describing the PET activity concentration σ(Φ) represents the conjunction of information from a *prior* probability distribution, ρ(Φ), and the *likelihood*, L(Φ), (34, 61) given by e.g.

(5) σ(Φ)=kρ(Φ)L(Φ)

where k is a constant. Equation 5 is similar to a Bayesian formulation of data integration. The prior probability distribution ρ(Φ) represents any information available about Φ independently from data (the reconstructed PET image in this case). This can be for example medical expert knowledge and/or information about modalities from other types of medical imaging data and biophysical prior information (41). The explicit choice of prior information is key to the use of the probabilistic method as will be discussed in detail later.

The likelihood L(Φ) is a probabilistic measure of how well a specific set of model parameters Φ explains the data, here the reconstructed PET image $\Phi_{\mathrm{PET}}$. In general, the likelihood represents uncertainty on data as well as imperfections in the physical model (modeling errors) (34). In the present case this leads to $L(\Phi )=\rho_{\Phi }(G\Phi )$ which can be trivially obtained using Eq. 3 in case the noise is multivariate Gaussian, and Eq. 4 in case the noise is multivariate log-normal. The likelihood provides a probabilistic measure of how good a specific Φ is in explaining the observed reconstructed PET image $\Phi_{\mathrm{PET}}^{\mathrm{obs}}$ according to the noise model. The likelihood should be chosen to represent these noise characteristics, as discussed previously, and as an example will demonstrate (see 3).

#### 2.1.1. Sampling from σ(Φ)∝L(Φ)σ(Φ)

A number of methods exist that allow sampling of a probability distribution, such as the posterior probability distribution σ(Φ) defined in Eq. 5, to provide a collection of realizations distributed according to the probability distribution (62–66). Most of these algorithms, such as the rejection sampler, the Gibbs sampler, and the Metropolis-Hastings algorithm, require that one can evaluate the posterior distribution for any given set of model parameters, σ(Φ) (38, 62, 63).

The extended Metropolis algorithm (67), a variant of the Metropolis-Hastings algorithm (62), can be used to sample the product of two probability distributions, such as here the prior ρ(Φ) and the likelihood L(Φ) in Eq. 5 (65). It can can be implemented as follows:

1. Generate an initial set of model parameters $\Phi_{\mathrm{current}}$ as a realization from ρ(Φ).
2. LOOP start
  a. **Exploration** Generate a set of model parameters $\Phi_{\mathrm{propose}}$ in the vicinity of $\Phi_{\mathrm{current}}$. Iterating only this “exploration” step must lead to sampling the prior ρ(Φ) through a random walk.
  b. **Exploitation** Accept the move from $\Phi_{\mathrm{current}}$ to $\Phi_{\mathrm{propose}}$ with a probability of
    (6) $$P_{\mathrm{acc}}=\min\left[1,\frac{L(\Phi_{\mathrm{propose}})}{L(\Phi_{\mathrm{current}})}\right].$$
    If the move is not accepted the Markov chain stays at $\Phi_{\mathrm{current}}$, otherwise the state is updated such that $\Phi_{\mathrm{current}}=\Phi_{\mathrm{propose}}$.
  c. **Store current state**, store $\Phi_{\mathrm{current}}$.
3. LOOP until enough realizations have been sampled

A benefit of the extended Metropolis algorithm is that neither σ(Φ) (as is needed for applying the classical Metropolis type algorithms (62, 63)) nor ρ(Φ) need to be evaluated, it is enough that an algorithm exists that can sample ρ(Φ), and that the likelihood L(Φ) can be evaluated for any set of model parameters Φ.

This is important in the current context, as this means that any algorithm that can generate a set of model parameters representing a priori knowledge about the *in vivo* distribution of activity concentration can in principle be used as prior information. No analytical description of the prior needs to be available. An analytical description may be available (such as when using the multivariate Gaussian prior), but still, using the extended Metropolis algorithm one must use a sampling method to perform a random walk in the prior, which for a multivariate model can be done using for example the computationally efficient sequential Gaussian simulation (48) or the computationally less efficient Metropolis-Hastings algorithm.

The requirement of proposing a new set of model parameters in the vicinity of the current set of model parameters, such that the prior is sampled, can be achieved by using, for example, the sequential Gibbs sampler, which essentially relies on performing a conditional simulation of a subset of the model parameters in an N-dimensional prior, conditioned on the rest of the model parameters (60, 68).

In the geostatistical community, many statistical methods have been proposed that allow sampling from statistical models representing various simple to complex structures. These can for example be based on 2-point Gaussian statistics (48), or more complex multiple-point statistical models inferred from sample images using either geostatistical simulation (49) or generative adversial networks (GANs) (69–71).

Many of these methods can by themselves, and combined, be used with the sequential Gibbs sampler in the exploration step of the extended Metropolis algorithm described above (60, 68–70, 72). As discussed and demonstrated in (65, 68, 72) this opens up the possibility of using many variants and combinations of such geostatistical simulation methods (49, 73), to describe rather complex information about expected spatial structures.

In practice, the first set of model parameters considered by the sampling algorithm is selected as a random realization from the prior model. Initially, the algorithm will search for an a priori acceptable set of model parameters that leads to a data fit according the noise model (as quantified by the likelihood). This is called the *burn-in phase*, at the end of which the Markov chain has reached *burn-in*. This is typically found when the likelihood value stabilizes around a certain level. The actual level is associated with the specific choice of noise model. When burn-in has been reached the Markov chain has converged, and the posterior distribution is being sampled, and each set of current model parameters will represent a realization of the posterior distribution. All sets of model parameters considered before burn-in are discarded, and all sets of model parameters (realizations) after burn-in represent a sample of the posterior distribution. See more details about running the extended Metropolis algorithm in (65, 72). No single approach exists that allows determining both if and when burn-in has been reached. In addition, it may be non-trivial to determine whether the Markov chain has converged and whether enough independent realizations have been obtained, as discussed by e.g. (74, 75).

To summarize, to infer information about the in situ activity concentration Φ from the reconstructed PET image $\Phi_{\mathrm{PET}}$, as a probabilistic formulated (deconvolution) inverse problem using the extended Metropolis algorithm, one needs 1) to select a prior model, from which realizations can be sampled through a random walk, 2) to be able to evaluate the forward problem (here in form of evaluation of the convolution GΦ, in Eq. 2), and 3) to be able to evaluate the likelihood L(Φ). Step 1) is required in the exploration step, and step 2) and step 3) in the exploitation step in the extended Metropolis algorithm.

The choices of G, L(Φ), and ρ(Φ) are problem specific, as also demonstrated below. G and L(Φ) are related to the choice of scanner and reconstruction method, while the choice of prior information, ρ(Φ), will depend highly on the type of tissue being scanned. In practice, this involves taking into account and quantifying prior information from medical experts.

## 3. Example applications

Some performance aspects of the algorithm were evaluated on PET images obtained using both a phantom experiment and in vivo data. Both PET images were acquired using a combined PET/MRI system (Siemens Biograph mMR) and a specific choice of PET reconstruction algorithm.

### 3.1. Data

#### 3.1.1. Phantom experiment

**Figure 1:**
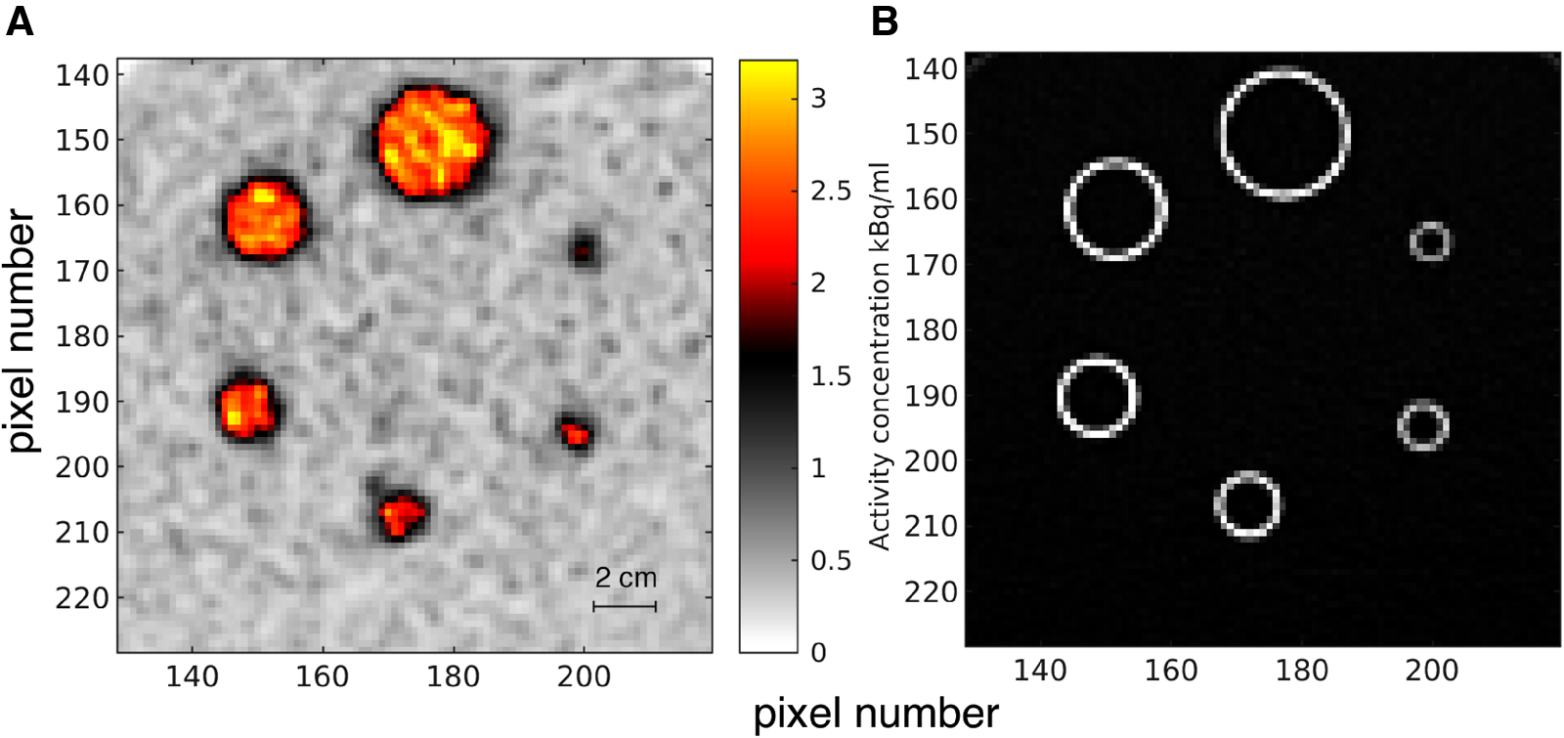
Phantom experiment. (**A**) Zoom of reconstructed PET image showing the spheres of the NEMA phantom, $\Phi_{\mathrm{PET}}^{\mathrm{obs}}$. Pixel size is 2.08×2.08 mm. (**B**) The corresponding CT image.

A phantom setup employed a body-mimicking National Electrical Manufacturers Association (NEMA) phantom (PTW, Freiburg, Germany). Briefly, a set of 6 hollow spheres and the background was filled with aqueous solutions of [^18^F]FDG. A low-dose CT (Siemens Biograph mCT) scan was used for CT-based attenuation correction. Reconstruction utilized 3D OP-OSEM with 3 iterations (i.e. using early stopping), 21 subsets, and a 4-mm Gaussian post-reconstruction filter. No resolution modeling was applied. Voxel size was 2.08×2.08×3.00 mm^3^. A single slice through the center of the spheres, zoomed to the central 90x90 voxels, was studied and shown together with a corresponding CT in Figure 1.

#### 3.1.2. In vivo data

**Figure 2:**
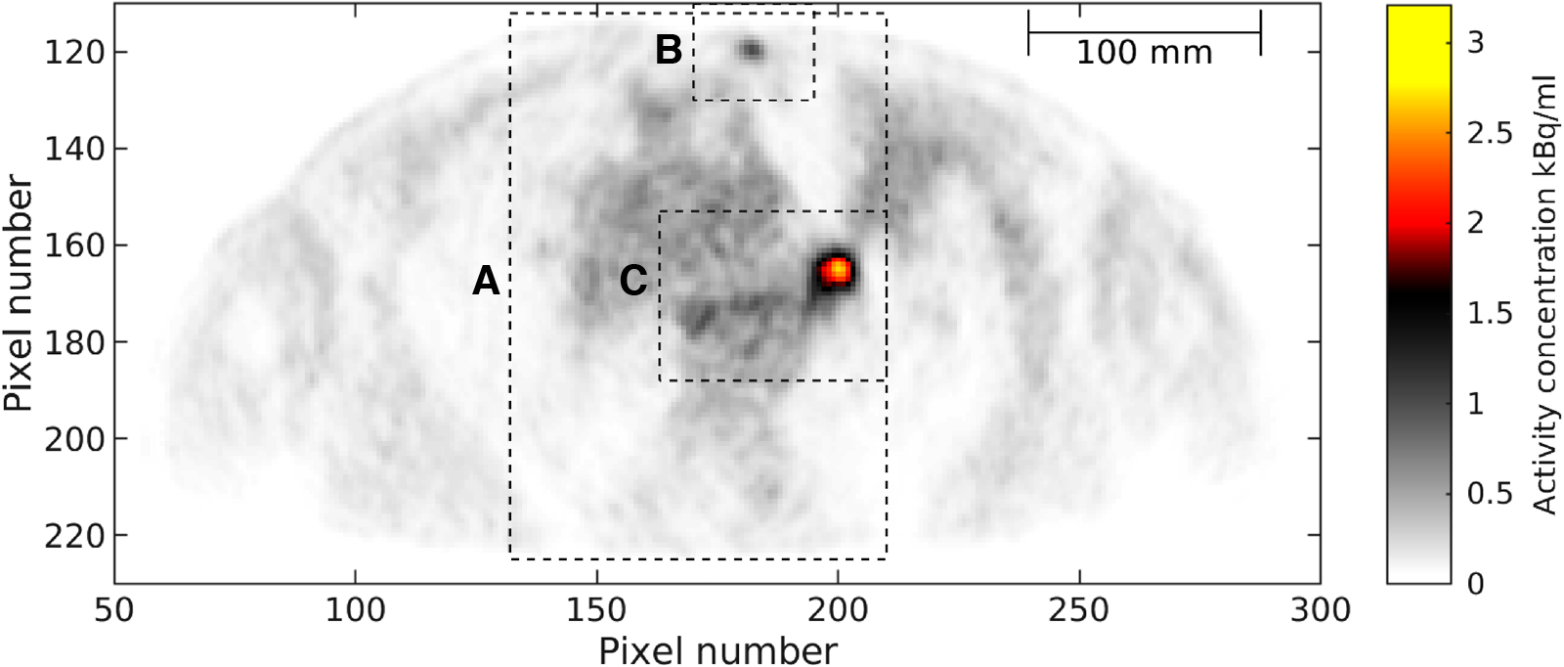
In vivo data. Reference PET image, $\Phi_{\mathrm{PET}}^{\mathrm{obs}}$. Pixel size is 2.08×2.08 mm. The rectangles indicate three areas, A, B and C, to be analyzed further.

An in vivo example was obtained from an ongoing study of patients with advanced non-small cell lung cancer. The study was approved by the departmental science committees at Rigshospitalet, by the Regional Ethics Committee, approval number H-3-2013-09, and by the Danish Data Protection Agency. The patient was scanned for 8 min, 60 min after injection of [^18^F] FDG (2 MBq/kg). The same reconstruction algorithm and parameters as used for the phantom case were used here, as well as a reconstruction employing PSF modelling and a 2-mm Gaussian post-reconstruction filter. A single slice through the hilar tumor and a subcutaneous metastasis was studied, and shown in Figure 2.

The goal is now to infer information about the tracer distribution Φ, from the reconstructed PET image, $\Phi_{\mathrm{PET}}^{\mathrm{obs}}$, using the described methodology, which requires the ability to evaluate the likelihood of a specific PET image, L(Φ), and the ability to sample PET images from a chosen prior distribution ρ(Φ).

### 3.2. The likelihood

Barrett et al. (32) demonstrate in a theoretical study how the pixel activity of a reconstructed PET image, $\Phi_{\mathrm{PET}}^{\mathrm{obs}}$, obtained using an EM reconstruction algorithm follows a multivariate log-normal distribution. This is equivalent to assuming a multivariate Gaussian model to describe the deviation between the reconstructed PET image, $\Phi_{\mathrm{PET}}^{\mathrm{obs}}$, and the convolved image, GΦ (Eq. 2), in the log image domain. Hence, the likelihood can be evaluated using Eq. 4 as

(7) $$L(\Phi )=\rho_{\Phi }(G\Phi )$$

Thus, evaluation of the likelihood, trough Eqs. 7 and 4, requires knowledge about the linear convolution operator G, and a covariance model, $C_{t}$, describing the covariance between pairs of pixels in the log image domain. Below we demonstrate how to empirically estimate G and $C_{t}$ by scanning a known object (the phantom) as suggested by e.g. (8, 56). As both considered PET images have been scanned using the same scanner using the same PET reconstruction algorithm, the same G is used. The type of noise model (a multivariate log-normal model) inferred from the phantom data is also used in both cases. But, as the local variance depends on the local signal level, the specific local magnitude of the covariance used will differ for the two cases. If another scanner is used and/or another type of reconstruction applied, then the noise model and the convolutional operator needs to be estimated anew.

#### 3.2.1. The convolution operator, G

The convolution operator G can be estimated by scanning a known object, such as the phantom in Figure 1A, by ensuring that the log-data residual

(8) $$n=\log(\Phi_{\mathrm{PET}}^{\mathrm{obs}})-\log(G\Phi_{ref})$$

is minimized. $\Phi_{ref}$ refers to the known phantom model. The point spread function is assumed to be described by a Gaussian type averaging kernel given by

(9) $$G_{ij}=k_{j}\exp(-h_{ij}^{2}/a^{2})$$

where $h_{ij}$ is the distance between sets of a centered pixel representing $\phi_{i}$, and other pixels, representing $\phi_{j}$, in the PET image, and a is the range that determines the width of the Gaussian averaging kernel. $k_{j}$ is a normalization that ensures that each row in G sums up to 1.

The optimal choice of the width of the averaging kernel a is obtained by evaluating n for a range of values of a from 0.0 to 10.0 mm in steps of 0.1 mm. a=4.4 mm, or 2.1 pixels, equivalent to a FWHM of 7.4 mm, leads to the lowest log-data residual, and is used from hereon.

#### 3.2.2. The noise model

**Figure 3:**
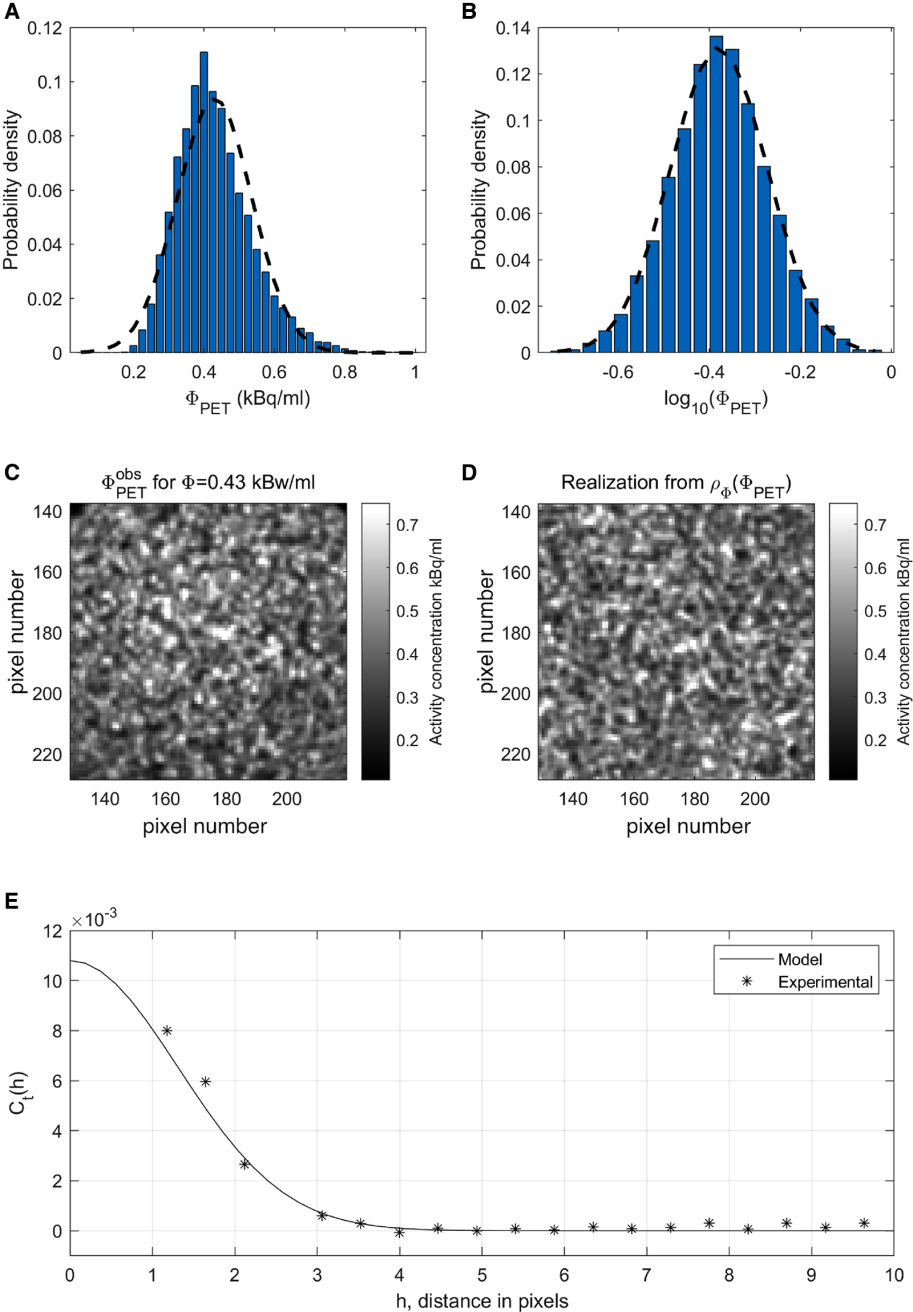
Inferring the noise model from data. (**A**) 1D histogram (blue) and best fit 1D normal distribution (dotted) of $\Phi_{\mathrm{PET}}^{\mathrm{obs}}$. (**B**) 1D histogram (blue) and best fit 1D normal distribution (dotted) of $\Phi_{\mathrm{PET}}^{\mathrm{obs}}$ in log image domain. (**C**) $\Phi_{\mathrm{PET}}^{\mathrm{obs}}$. (**D**) A realization of the inferred noise model $\rho_{\Phi }(\Phi_{\mathrm{PET}})$ for activity level Φ=0.43 kBq/ml. (**E**) Experimental and modeled covariance in the log image domain.

Figure 3A shows a histogram (blue) of a 2D slice of the activity concentration in a reconstructed PET image $\Phi_{\mathrm{PET}}^{\mathrm{obs}}$ (shown in Figure 3A) in an area of known constant low (0.43 kBq/ml) activity concentration. The 1D distribution is skewed, and a best fitting 1D normal distribution (dashed line in 3A), does not represent the histogram well. Figure 3B shows the corresponding histogram in the log image domain, i.e. the histogram of $\log(\Phi_{\mathrm{PET}}^{\mathrm{obs}})$, as well as the best fitting normal distribution (dashed line). The findings are consistent with the expectation that log-normal 1D distribution is a good representation of the pixel variability in $\Phi_{\mathrm{PET}}^{\mathrm{obs}}$ (32), and hence justifies the use of the log-normal model for the likelihood function.

In addition, the noise variance depends on the local mean activity concentration (32). The standard deviation of the noise has been estimated in the log image domain for the low and high activity concentration levels, around Φ=0.43 kBq/ml (from Figure 3C) and Φ=2.77 kBq/ml (within the big spheres in Figure 1A), and has been estimated at $\mathrm{std}(\log_{10}(\Phi_{\mathrm{PET}}^{\mathrm{obs}}))=[0.1060,0.0402]$. As expected the relative noise level is higher in the areas of low count and low activity, and lower in areas of high activity (32). The standard deviation of the noise in the log image domain for any activity level is estimated using simple linear interpolation between the two considered activity concentration levels. For Φ≤0.43 kBq/ml the absolute noise level is assumed constant, both because the activity concentration estimate will be based on very few data, and also because, for the clinical case of cancer considered here, the focus on the case with in vivo data is the higher rather than lower activity levels. This may differ among different clinical situations.

From Figure 3C it is evident, as also reported by (32, 52), that some correlation between neighboring pixels exists, and hence it is assumed that the noise can be described by a correlated spatially isotropic 2D Gaussian probability distribution in log image domain $N(d_{t},C_{t})$, where $d_{t}$ is the mean activity concentration, and $C_{t}$ the covariance of the data residual in log image space.

Figure 3E (stars) shows the experimental covariance in log image space computed from $\Phi_{\mathrm{PET}}^{\mathrm{obs}}$ in Figure 3C, assuming $d_{t}=0.43$ kBq/ml, as well as the best fitting Gaussian type covariance model with an isotropic range of 1.85 pixels and a variance of 0.01 (a standard deviation of 0.1) in log-space, equivalent to a full width at half maximum (FWHM) of 6.4 mm. For this analysis, we relied on classical semivariogram analysis, as described in e.g. (73).

Figure 3D shows one realization of the inferred noise model, at the same low activity concentration level, $d_{t}=0.43$ kBq/ml, as in Figure 3A. This realizations appears to have a spatial distribution similar to the observed noise, Figure 3A, which suggests that the chosen noise model does reflect the actual noise well, for this specific signal level.

As the noise model is not linear in the signal level, ideally one must construct and invert $C_{t}$ in each iteration of the Monte Carlo simulation (as the current set of model parameters changes slightly at each iteration). This is however computationally demanding. Instead, we suggest smoothing the PET image data, using a simple 4×4 pixel moving average, from which a linear noise model is constructed as described above, with the local variance in the log image domain following the average signal value. In order not to underestimate the noise due to the use of this linear noise model (and hence risk overfitting), the noise variance is increased by 20%. Using a linear noise model, $C_{t}$ needs only be constructed and inverted once, providing a more efficient sampling. With this approach the same type of likelihood (Eq. 7) can be used for both the phantom and in vivo data, but, as the magnitude of the two reconstructed PET images differs, so will the specific choice of $C_{t}$.

The estimated $C_{t}$ (scaled by the local average activity concentration) and linear forward operator G is used in the remainder of the article. Based on this information the likelihood (Eq. 7) can be computed, which allows evaluating the “exploitation step” in the extended Metropolis algorithm.

### 3.3. Prior information

The method presented above requires realizations of the prior probability density can be simulated using a sampling algorithm. By construction, the realizations that are generated by such an algorithm then represent the available prior information. The process of quantifying prior information is in practice a two-step process. First, a medical expert describes known (a priori) information. Then, a statistical model is chosen that best reflects information from the medical expert. Several realizations of this statistical model are then generated and visualized and validated by the medical expert. This process is iterated until the prior model generator is deemed appropriate by the medical expert.

The prior model is, thus, not based on an implicit mathematical model (in fact, no mathematical model is needed to describe the prior) but is instead an explicit choice, guided preferably by medical expertise, that can be visualized, analyzed, and validated independently of the PET image data.

Below two such prior models are constructed to reflect prior knowledge related to the phantom and in vivo cases.

#### 3.3.1. A priori model for the phantom data, $\rho_{1}(\Phi )$

Everything is in principle known about the activity concentration relating to the PET image data in Figure 1. It should consist of 6 perfect circles, in different sizes with their outline as imaged in the CT image, Figure 1B. The activity concentration is expected to be constant and high within the spheres and constant and low outside the spheres.

As an example, the following prior information is considered: The real activity concentration distribution is discrete and bimodal, and the low and high activity concentration is assumed to be within [0.1,1.1] and [2.1,3.2] kBq/ml respectively. In both cases, a uniform distribution is used to represent the activity concentration level. The spatial distribution of the areas of high and low activity concentration is assumed to follow a truncated 2D multivariate normal distribution, based on a 2D Gaussian type isotropic covariance model with a range of 10 pixels. Truncation is done such that the smallest 9% realized pixel values are associated with high activity and the rest with low activity.

**Figure 4:**
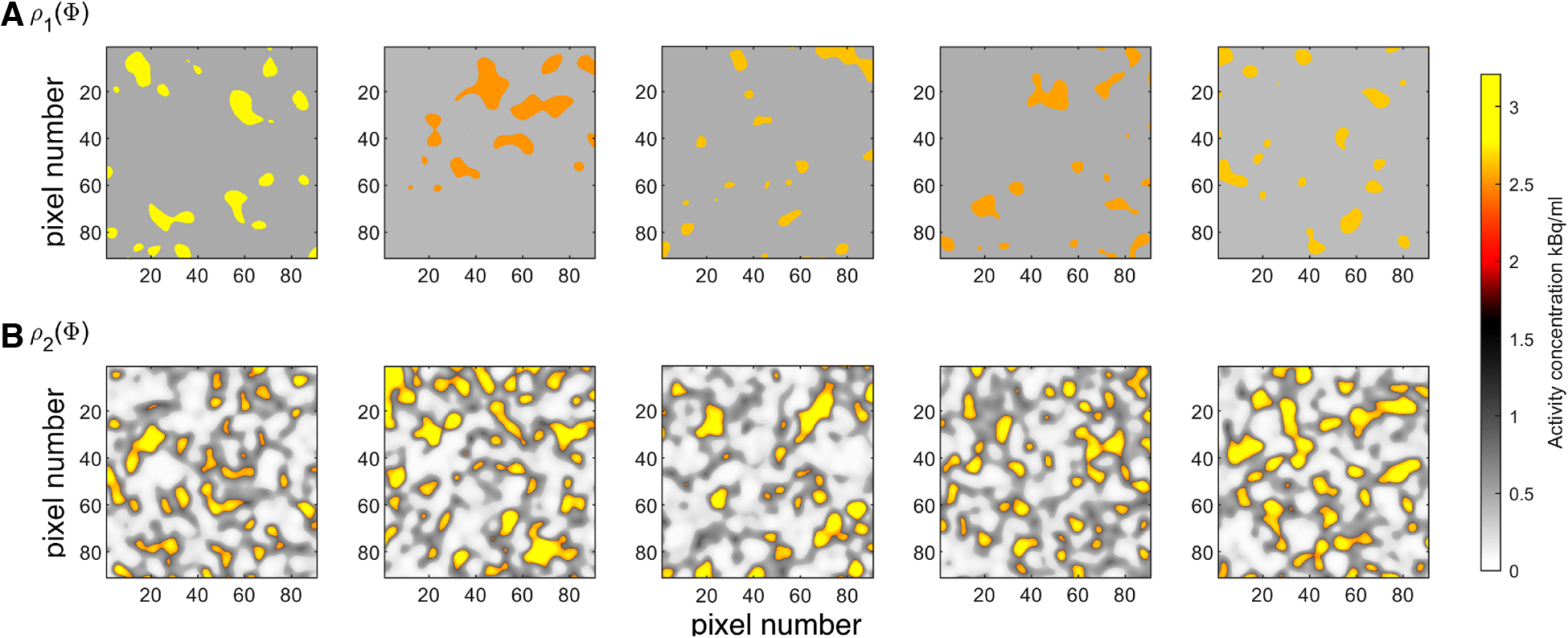
Five realizations from the two considered prior models (**A**) $\rho_{1}(\Phi )$, and (**B**) $\rho_{2}(\Phi )$. Colorscale as in Figure 1A.

**Figure 5:**
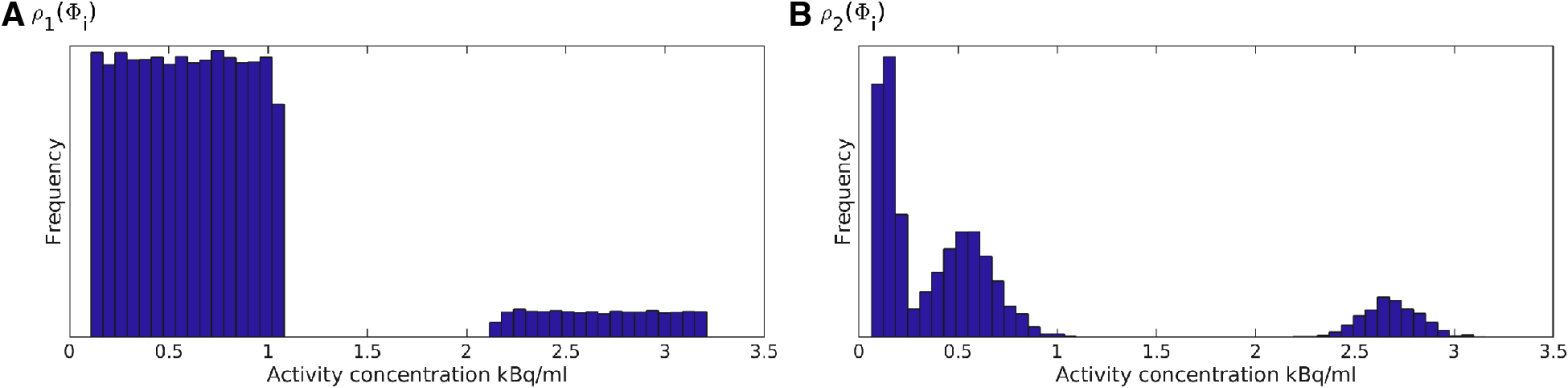
A priori 1d marginal distribution from (**A**) $\rho_{1}(\Phi )$, and (**B**) $\rho_{2}(\Phi )$.

In practice, a realization of such a model can be generated by first generating a realization of the multivariate normal distribution with unit variance followed by truncation, such that all values above −1.34 (the 9% quantile of the normal distribution) will refer to the low-activity region, and all values below will refer to the high-activity region. See e.g. (48) for description of several Gaussian based simulation methods. Then, each region is populated with an activity value realized from the two uniform distributions. The resulting set of model parameters will be a realization of the prior model as defined by the described algorithm. An analytical formula does not exist to describe this prior model, but in any case, it needs never be evaluated using the proposed method. It is enough that an algorithm exists that can sample from the prior model. Figure 4A shows 5 realizations of the prior model $\rho_{1}(\Phi )$, and Figure 5A the corresponding 1d marginal distribution of activity concentration, $\rho_{1}(\Phi_{i})$.

#### 3.3.2. A priori model for the in vivo data, $\rho_{2}(\Phi )$

The PET image obtained by scanning near a lung, Figure 2, represents a real in vivo case. The following observations were made and reflect prior information:

∙ I1, Healthy tissue is expected to be associated with low uptake.
∙ I2, Cancer lesions are expected to be associated with high uptake.
∙ I3, The uptake can vary between and within tumors.
∙ I4, The boundary between regions with and without cancer, is expected to be relatively sharp.

The prior model representing the phantom case, $\rho_{1}(\Phi )$, is too simple to represent this type of prior information. To construct a more realistic prior resembling the available expert information, I1-I4, a multivariate normal distribution with a Gaussian type covariance model with a range of 8 pixels is assumed, but with a trimodal 1D marginal distribution, describing three types of tissue, $t_{1}$, $t_{2}$, and $t_{3}$, is defined as:

$t_{1}$: Cancer lesions. Homogeneous regions with activity concentration following a Gaussian distribution with mean 2.7 kBq/mL and standard deviation of 0.13 kBq/mL, i.e. $N(2.7,0.13^{2})$.
$t_{2}$: Tissue, type A; e.g. representing physiologic uptake in muscles and mediastinum. Intermediate activity concentration following a Gaussian distribution with mean 0.55 kBq/mL and standard deviation 0.15 kBq/mL, i.e. $N(0.55,0.15^{2})$.
$t_{3}$: Tissue, type B; e.g. representing physiologic uptake in lung parenchyma. Low activity concentration, uniform in the interval from 0.06 to 0.2 kBq/mL.

This prior is referred to as $\rho_{2}(\Phi )$, from which 5 realizations are shown in Figure 4B, and the 1D marginal, $\rho_{2}(\Phi_{i})$, is shown in Figure 5B. The units on the axes in Figure 4 is pixels of size 2.1×2.1 mm^2^, as given in the PET images, Figures 1 and 2, but the resolution used in the prior space of activity concentration is 4 times finer (pixel size 0.25×0.25 mm^2^).

Several specific choices need to be made, especially setting up $\rho_{2}(\Phi )$. Any of these choices could, and should, be debated between experts in the field (clinical experts and physicians), in practice by analyzing realizations of the resulting prior, as seen in Figure 4*B*. The prior model $\rho_{2}(\Phi )$ is constructed to represent the prior information available in the in vivo case and is not a general prior model intended to be used for other cases.

The two considered prior models, $\rho_{1}(\Phi )$ and $\rho_{2}(\Phi )$, represent two explicit choices of a prior model. Any results and analysis presented below should be considered relative to the a priori assumptions as visualized in Figure 4.

## 4. Results - phantom experiment

Both prior models are consistent with what is known a priori about the phantom case, in the sense that the real tracer distribution is a possible realization of both $\rho_{1}(\Phi )$ and $\rho_{2}(\Phi )$, while the former is more informed than the latter. Therefore, as an example, both prior models are considered to analyze the PET image phantom data.

**Figure 6:**
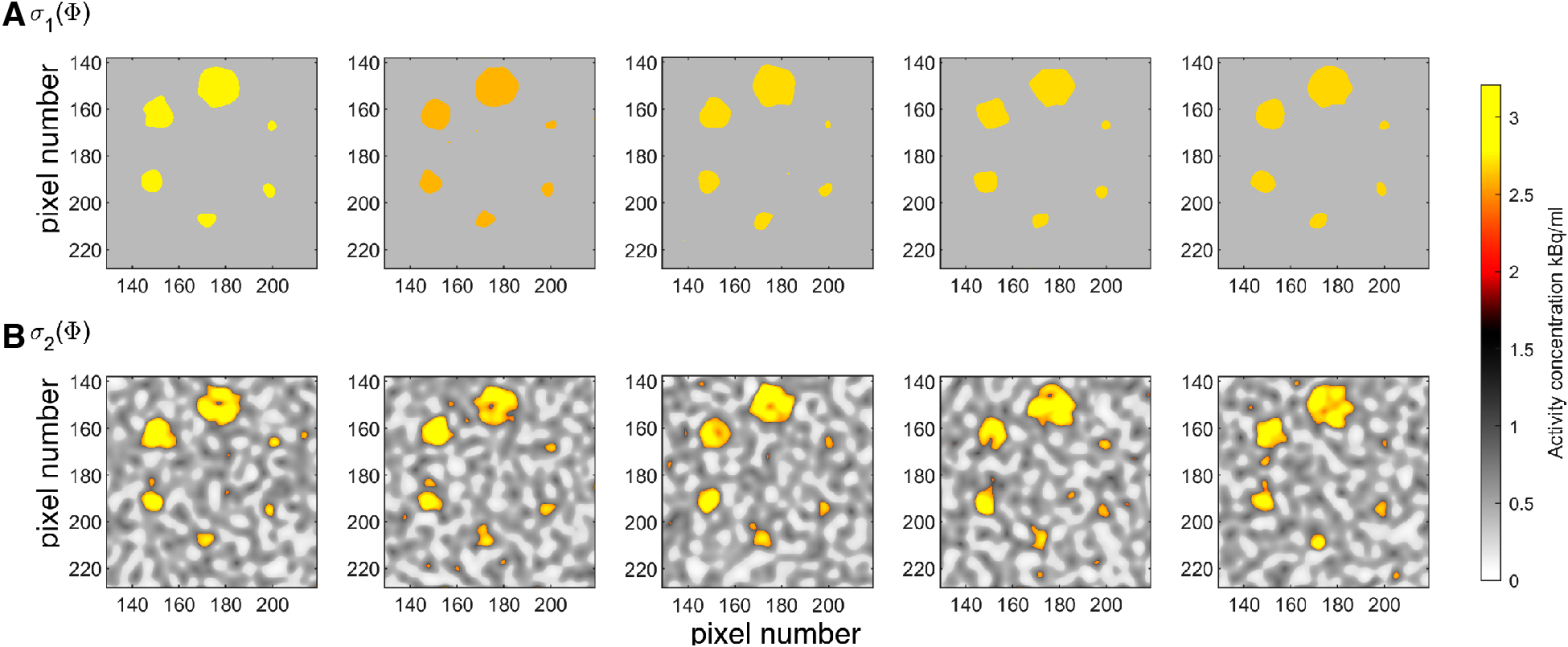
Five realizations from the posterior distributions (**A**) $\sigma_{1}(\Phi )$, and (**B**) $\sigma_{2}(\Phi )$ related to the prior distributions $\rho_{1}(\Phi )$ and $\rho_{2}(\Phi )$. Colorscale as in Figure 1A.

Figure 6 shows 5 realizations (out of 385 generated) of the posterior probability distribution, obtained by running the extended Metropolis algorithm, using each type of prior model. Sampling is initiated from an independent realization of the prior. Burn-in is reached at around 15,000 iterations, and the Markov chain is run for 400,000 iterations. One independent realization is obtained for around every 1,000 iterations after burn-in, which is identified using autocorrelation analysis as discussed in (65).

The posterior distribution refers to the conjunction of all available information, and each of the presented realizations is consistent with both the prior model (compare to Figure 4), the physics (smoothing), and the assumed noise model. The variability within the whole collection of realizations represents the combined uncertainty. Hence, the probability of a certain event occurring is proportional to the frequency with which it occurs in the posterior sample, Figure 6 (76). This allows a quantitative approach for the analysis of the in situ activity concentration. An event could, for example, be E: “Pixel A has high activity”, or E: “Pixel A and Pixel B are connected by a coherent region of high activity.”

A simple visual inspection of the realizations of the posterior, Figure 6, reveals that the correct location and size of the spheres can be seen in most realizations, which suggest they are well resolved. It also suggests that the resolution of $\sigma_{1}(\Phi )$ is higher than that of $\sigma_{2}(\Phi )$. This is simply related to the fact that the information content of $\rho_{1}(\Phi )$ is higher than that of $\rho_{2}(\Phi )$. $\rho_{2}(\Phi )$ allows some correlated spatial variability within both regions of high and low activity, which can be seen, as expected a priori.

### 4.1. Analysis of the sample from the posterior

**Figure 7:**
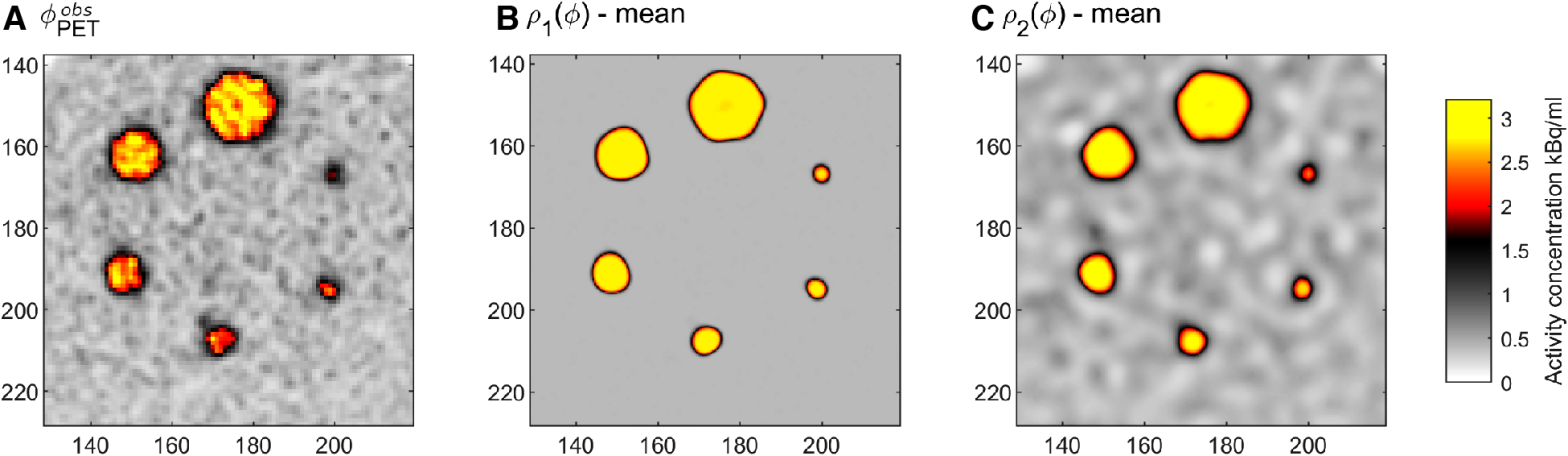
(*A*) Reconstructed PET image, $\Phi_{\mathrm{PET}}^{\mathrm{obs}}$, as Figure 1A. Point-wise mean tracer activity from (**B**) $\sigma_{1}(\Phi )$, and (**C**) $\sigma_{2}(\Phi )$.

Several statistical properties of the posterior can now be computed, which can be useful for decision-making for a medical expert. The simplest measure is the point-wise mean activity concentration level, as shown in Figures 7B,C for both $\rho_{1}(\Phi )$ and $\rho_{2}(\Phi )$. These images can be compared to the original PET image data, Figure 7A, and provides, in comparison, a sharper image with less noise and more accurate activity concentration levels, as will be analyzed below. Notice that no ringing effects often referred to as Gibbs ringing are noticeable near the sharp boundaries of the spheres. Such ringing effects are often visible when applying deconvolution methods, (17). The low amplitude correlated features in the low activity region in Figure 7C is due to the correlated features of $\rho_{2}(\Phi )$.

**Figure 8:**
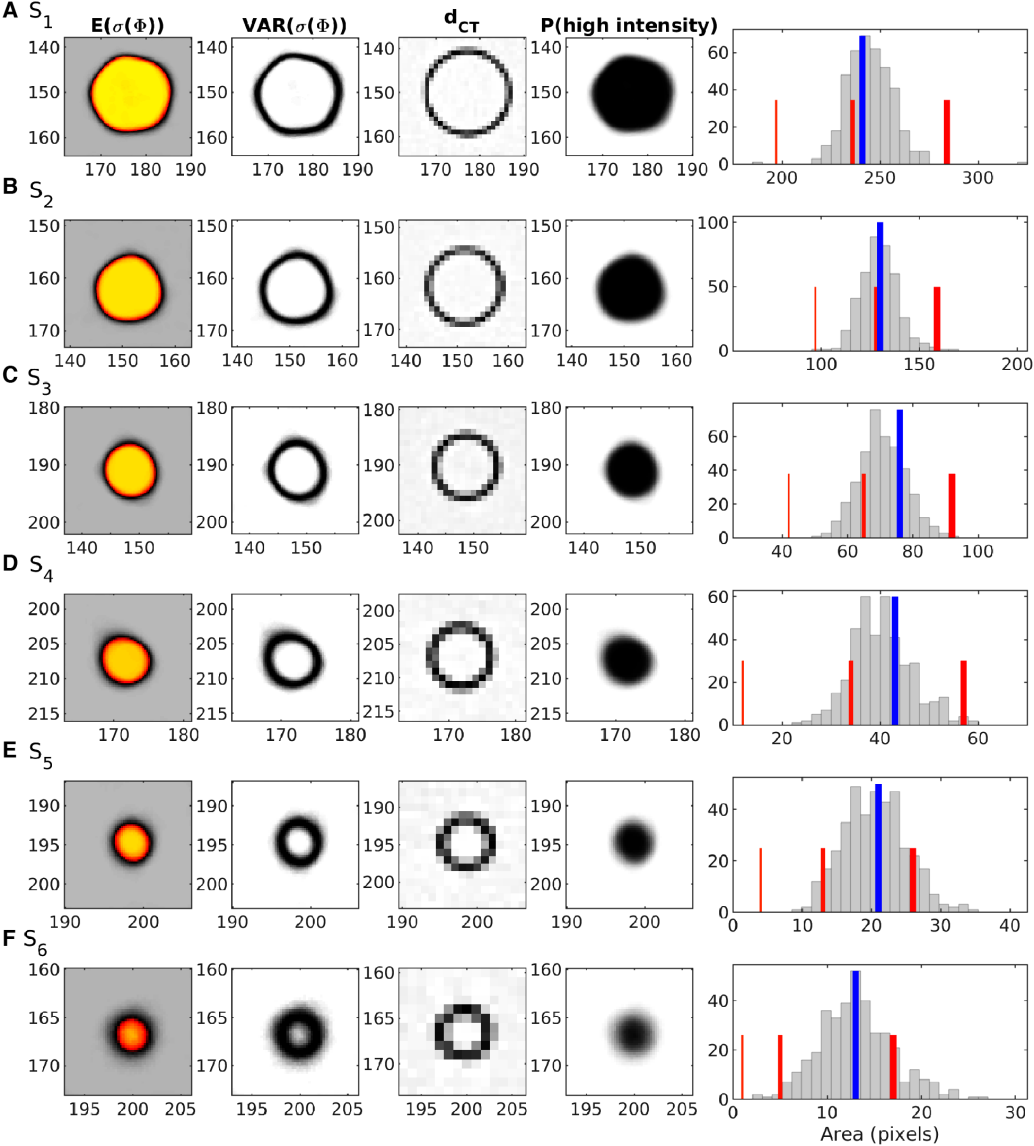
Statistics from $\sigma_{1}(\Phi )$, around the 6 spheres ($S_{1}$–$S_{6}$). Column 1: Pointwise mean activity (colorscale as in Figure 1A). Column 2: Pointwise variance (black:high, white:low). Column 3: CT data. Column 4: Posterior probability of having high activity (>1.5 kBq/ml) (black:1, white:0). Column 5: Posterior probability of the area of the region with high activity (>1.5 kBq/ml). The blue line indicates the area obtained from the CT image. Red lines indicate areas obtained by thresholding the reconstructed PET image at levels >1.0 (thick), >1.5 (medium), and >2.0 kBq/ml (thin).

Figure 8 present posterior statistics (in case considering $\rho_{1}(\Phi )$ as prior information) around each of the 6 spheres. The first column shows the pointwise mean, and the second column the pointwise variance of 400 realizations. The third column shows the corresponding part of the CT image, and it is clear that the location of high variance in column two corresponds closely to the edges of the spheres as imaged in the CT image in column three. The fourth column shows the pointwise probability that the activity concentration is higher than 1.5 kBq/ml, P(Φ>1.5 kBq/ml). This particular threshold value is chosen as it marks a clear split between low and high activity regions, as illustrated on the 1D marginal distribution in Figure 5A. The last column shows the posterior distribution of the area of the coherent set pixels with activity concentration above 1.5 kBq/ml, found by counting the number of high-activity pixels in a connected region around the center pixel in all obtained realizations of the posterior. The inner area of the circles in the CT image is shown by the blue line (which is used as a reference for comparison). The red lines reflect the area of coherent high activity, using simple thresholding on the reconstructed PET image. Neither the mean estimate (column 1) or the probability of high activity (column 4) shows perfect circular shapes (as the real phantom has), due to the noise on the reconstructed PET image. However, a key feature in Figure 8 is that the posterior uncertainty is high at the transition between high and low activity, where the imperfections in the circular shapes are visible. The inner (high activity) and outer (low activity) regions are very well resolved.

Figure 8 column 5 demonstrates that simple thresholding to obtain the area of high activity is problematic. If a low threshold is used (>1.0 kBq/ml, thick red line) the area is overestimated for larger spheres. If a medium threshold is used (>1.5 kBq/ml, medium-thick red line) the area is well estimated for larger spheres, but underestimated for smaller spheres. If a realistic threshold for the expected activity is used (>1.5 kBq/ml, thin red line) the area is underestimated for all sphere sizes. These effects are related to the smoothing and damping of the amplitude of small high activity regions in the reconstructed PET image discussed previously.

Figure 8, and the equal tailed 95% credible interval in Table 1, shows that the area of the spheres computed from the CT image is very consistent with the posterior distribution of the area of the spheres obtained from $\sigma_{1}(\Phi )$, as it is well within the 95% credible interval.

**Table 1:** Row 1–3) 2.5, 50, and 97.5% quantile of the posterior distribution of the area (in number of pixels) of high activity. Row 4) The inner area from the CT image. See also last column in Figure 8.

	$S_{1}$	$S_{2}$	$S_{3}$	$S_{4}$	$S_{5}$	$S_{6}$
Area $p_{0.025}$	223	113	58	29	12	6
Area $p_{0.500}$	244	129	70	40	21	13
Area $p_{0.975}$	266	150	85	53	30	22
Area from CT	241	130	76	43	21	13

Table 2 contain statistics related to the posterior distribution of the activity within each sphere. The first row lists the probability that the center location of the sphere has high activity (Φ>1.5 kBq/ml). The following three rows list some quantiles of the average activity,$\Phi_{\mathrm{av}}$, incoherent high-activity regions in the posterior sample. For all spheres the $p_{0.025}$ quantile is above 2.47 indicating a 95% probability that $\Phi_{\mathrm{av}}>2.47$ kBq/ml. This in contrast to the activity within the spheres obtained from the PET image, is below 2.0 kBq/ml for the three smallest spheres.

**Table 2:** Posterior statistics on the median activity within each sphere.

	$S_{1}$	$S_{2}$	$S_{3}$	$S_{4}$	$S_{5}$	$S_{6}$
P(Φ>1.5 kBq/ml)	1.0	1.0	1.0	1.0	0.99	0.94
Activity $p_{0.025}$	2.56	2.50	2.48	2.47	2.47	2.47
Activity $p_{0.500}$	2.76	2.73	2.72	2.67	2.69	2.68
Activity $p_{0.975}$	2.88	2.88	2.88	2.86	2.88	2.88
Activity from $\Phi_{\mathrm{PET}}^{\mathrm{obs}}$	2.46	2.28	2.02	1.76	1.51	1.30

Row 1) Probability of locating high activity at the center of the known position of the sphere. Row 2-4) 2.5, 50, and 97.5% quantile of the distribution of the mean activity (in kBq/ml) within the six spheres (when high activity identified). Row 5) median activity as measured directly in the reconstructed PET image $\Phi_{\mathrm{PET}}^{\mathrm{obs}}$.

To summarize, Table 1 and Figure 8 suggest that the estimated areas (and associated uncertainty) of high-activity regions are consistent with the actual area, of the particular 3 mm slice of the 2D phantom. Table 2 suggests that the posterior median activity and the credible intervals for the activity within each sphere are consistent with the expected high activity levels. The posterior mean activity images in Figures 7 and 8 provide a sharper, less noisy, image of the activity concentration than the observed $\Phi_{\mathrm{PET}}^{\mathrm{obs}}$ in Figure 1. Also, uncertainty analysis is readily available, and coherent sets of high-activity regions can be assigned with a probability of existence. The area and activity of these regions can also be quantified through a probability distribution, which in this case provided results consistent with the used reference phantom model.

## 5. Results - in vivo

For the analysis of the in vivo PET image, Figure 2, only the prior model representing the three tissue types, $\rho_{2}(\Phi )$, is considered, as the discrete bimodal nature of $\rho_{1}(\Phi )$ is expected to be inconsistent with the observed PET image data. Figure 2 shows the outline of three data subsets, A, B, and C, that will be considered below.

**Figure 9:**
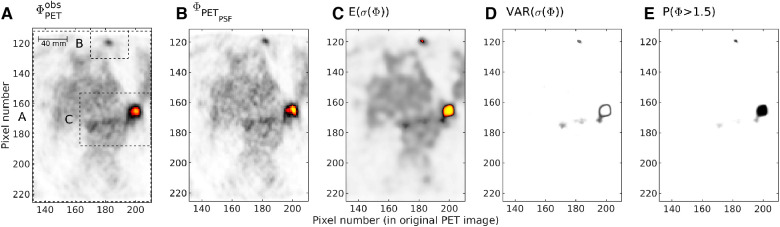
Subset A, (**A**) $\Phi_{\mathrm{PET}}^{\mathrm{obs}}$. (**B**) Fobs PET using PSF. (**C**) Point-wise posterior mean. Colorscale in (**A**), (**B**) and (**C**) as in Figure 1A). (**D**) Point-wise posterior variance (white:low, dark:high). (**E**) Point-wise probability of high activity (white:0, black:1).

Figure 9 shows the posterior statistics obtained considering the subset data set A. Figure 9A shows the reference PET image data in subset A, $\Phi_{{\mathrm{PET}}_{A}}^{\mathrm{obs}}$. This is equivalent to subset A in Figure 2. For comparison, Figure 9B shows the PET image data reconstructed with PSF modelling. Figures 9C,D show the pointwise mean and variance of the sample obtained from the posterior distribution $\sigma (\Phi_{A})$. As tissue type $t_{1}$ represents a high-activity cancer lesion, the probability of locating cancer can be quantified by computing the probability that the activity concentration is above 1.5 kBq/ml. This can trivially be computed from the obtained sample of the posterior and is shown in Figure 9E.

As expected, using a PSF in the PET reconstruction lead to slightly higher estimates of activity concentration, Figure 9B, than when no PSF was used, Figure 9A. Comparing the pointwise mean of the marginal posterior, Figure 9C, to both the PET image image obtained without and with a PSF, Figures 9A,B, it is clear that some of the noise has been suppressed, and that at the same time sharper structures can be identified, especially around the regions of interest, regions B and C, which are the only areas in which high intensities are present, Figure 9D. Figure 9D reveals that most of the uncertainty is related to the location and activity concentration of the boundary of the high-activity regions.

**Figure 10:**
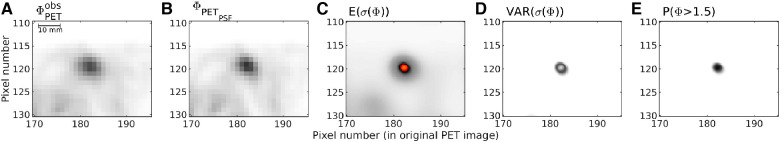
As Figure 9, but for subset B.

Figure 10 shows the same statistics as Figure 9, but only for data subsets B, to focus on the details. Each subset has been treated with a separate run of the proposed method, only in the specific subset. This allows using an even smaller pixel size of the model parameters, here 0.5×0.5 mm (1/4 of the pixel size of $\Phi_{\mathrm{PET}}^{\mathrm{obs}}$), and will provide similar results as using the full data subset A, except in a small region at the boundary where correlations exist due to the correlations in the prior, noise model, and G. As discussed previously, the choice of parameterization (the pixel size) provides an upper limit of the resolution one can expect and is here chosen small enough, that the resolution limit is controlled by the available information, and not the parameterization.

Figure 10D illustrates that the uncertainty is limited to the boundary of the high-activity lesion (that shows high posterior variance), but that the centre of the lesion is well resolved, with an apparent high high, as also evident in Figures 10C,E.

**Figure 11:**
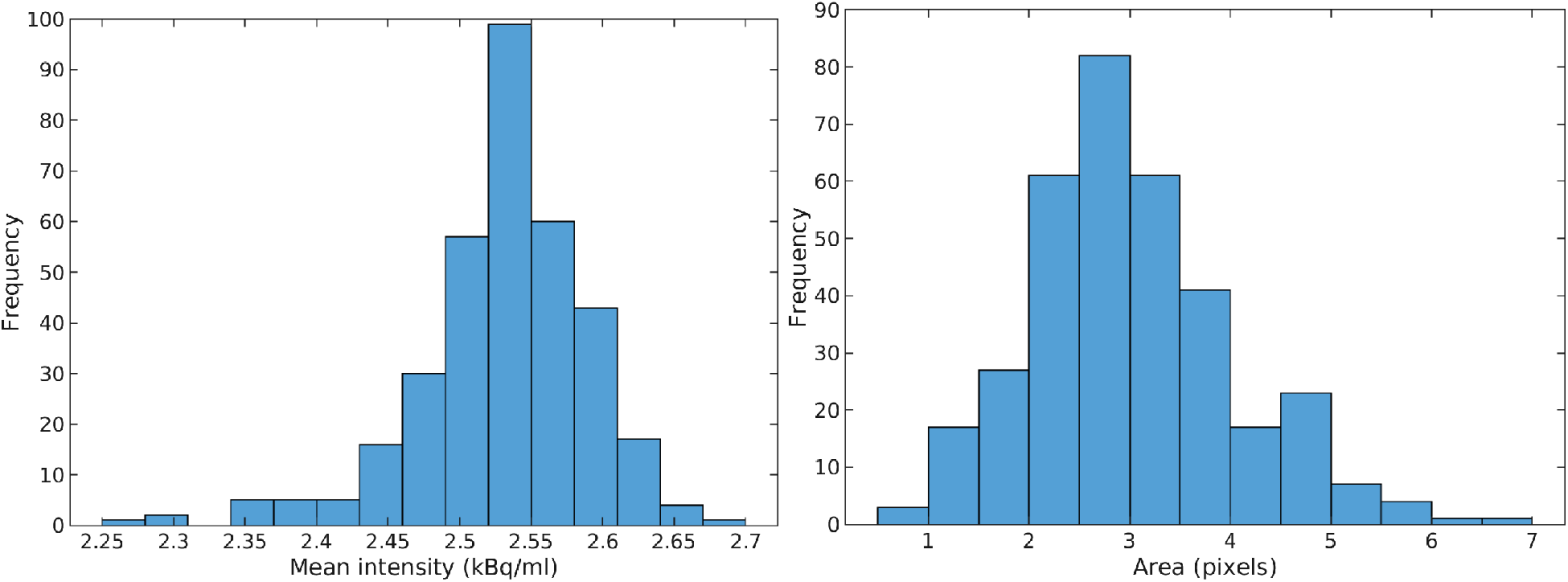
Mean activity (left) and area (right) of potential high activity cancer lesion at the center of data subset B.

Further, the size of the high-activity cancer lesion can be computed from each obtained realization, which can produce a posterior distribution of the size of the lesion in subset B as given in Figure 11A. These results suggest, that not only can a small cancer lesion (with an area between 1 and 7 pixels, each of size 2.2×2.2 mm) be resolved, the actual size and activity can potentially be quantified as well.

**Figure 12:**
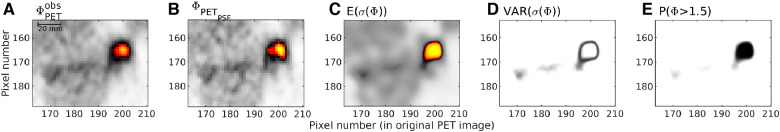
As Figure 9, but for subset C.

**Figure 13:**
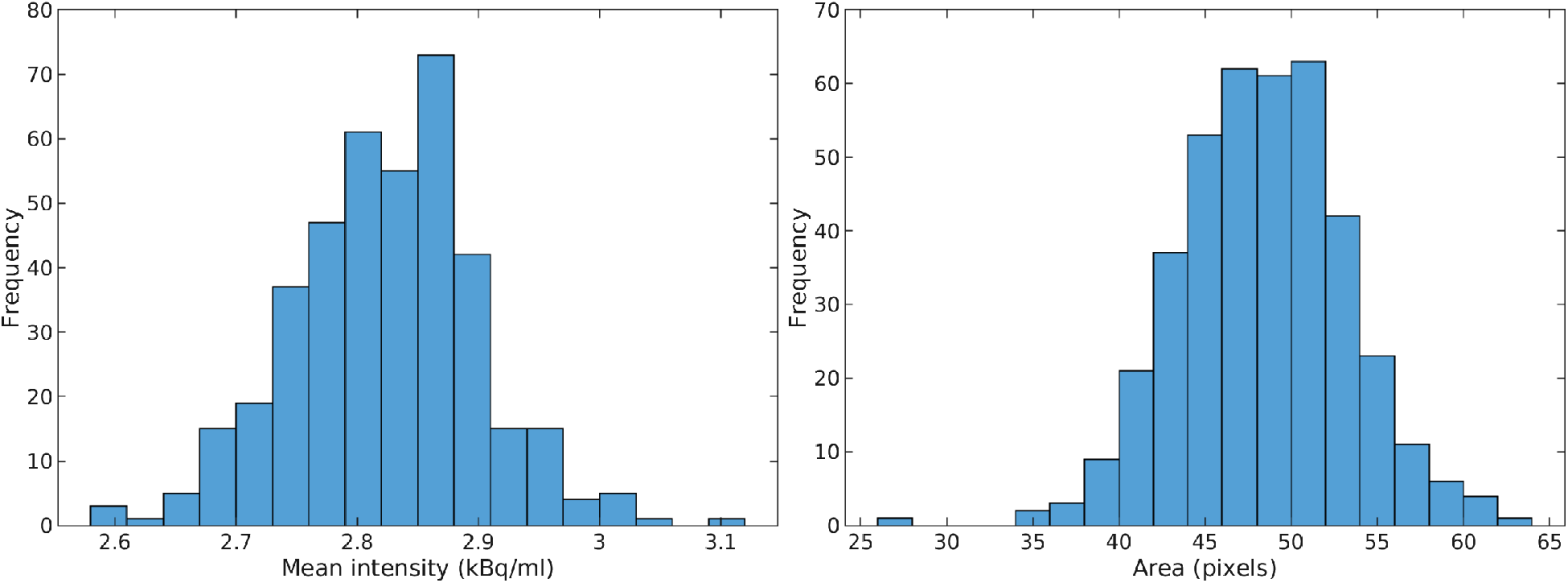
Mean activity (left) and area (right) of potential high activity cancer lesion at the center of data subset C.

The same analysis has been performed in data subset C, and the results are shown Figures 12 and 13. For data subset C, Figure 12A, the larger potential cancer lesion can be resolved quite well. The area and average activity can also be quantified as presented in Figure 13. The uncertainty is again limited to the boundary of the lesion, with a width of only about 1 pixel (in the original PET image) or 2.2 mm, Figure 12B. The two areas of relatively high activity to the left of the large lesion, are not resolved with respect to representing high activity (cancer) or not. They can represent high-activity lesions but only with a probability of around 0.1, Figure 12D.

The actual accuracy of the results obtained for this in vivo case, cannot be validated as the real in situ activity concentration is not known. The results indicate that using an informed prior model (given that the inferred noise model and smoothing operator are reasonable) could lead to both increased resolution, lower noise, and the possibility of informative quantitative analysis of the posterior distribution.

## 6. Discussion

A probabilistic approach for analysis of PET tracer activity concentrations from a PET image has been proposed, employing a noise model, a linear convolutional operator, and the use of explicit prior information. The approach was demonstrated on phantom and in vivo data using PET images obtained from a specific scanner (Siemens Biograph mMR) using a specific PET reconstruction method (OP-OSEM).

The method can be used to construct an enhanced image of the activity concentration distribution. Earlier methods for image enhancement based on partial volume or PSF correction (11, 12, 18) are in general associated with a trade-off between increased resolution or reduced noise. The early results shown here indicate that our method could be capable of both increasing resolution and decreasing noise, without producing artifacts such as Gibbs ringing, see e.g. Figure 9A vs 9B.

The full potential of the proposed method, though, should be realized by exploiting its properties as a probabilistic approach for image analysis. The probabilistic approach to image analysis of Eq. 5 has been considered in a number of cases (41), both for image restoration (36, 38) and image reconstruction (5, 35, 41, 46). However, in most cases the assumed prior information has been quite simple or based on a specific choice of mathematical model (38, 46). Also, most previous works have computed a statistical property of the posterior distribution, such as the model with maximum posterior probability $\Phi_{\mathrm{MAP}}$ (The MAP solution) (7, 12, 13, 41, 77). Often. in medical imaging the term “Bayesian” or “probabilistic” solution is used to describe the MAP solution (16). However, such a single set of model parameters will not in general be a representative realization of σ(Φ), and will not allow uncertainty analysis (34, 46).

Sampling methods, for a full sampling of the posterior, have also been considered in some cases for a specific choice of prior (46, 78) for both image reconstruction and restoration. Sampling of the posterior enables a probabilistic statistical analysis of the PET activity concentration (such as inferring information about the size and activity of potential cancer lesions, if the prior allows this), and is not limited to a particular statistical property such as the MAP. Our analysis was possible due to the use of probabilistic analysis with an explicit choice of prior, which was designed specifically to represent available knowledge from a medical expert.

In summary, our method differs from previous approaches, and in particular, MAP-based approaches, by 1) sampling the full posterior probability distribution, and 2) making use of any prior model (that can be sampled).

### 6.1. The explicit choice of a prior model

A key property of the proposed method is that it relies on, and requires, an explicit quantification of a medical expert’s prior information. The only requirement to the prior is that an algorithm exists that can sample from the prior through a random walk. The limit to what prior assumptions can be taken into account is therefore only limited by the capabilities of available simulation methods. Several simulation methods developed with the specific aim of reproducing spatial patterns with various complexity have been developed in the field of geostatistics (48, 49), and can readily be utilized (and combined) in the current workflow (60, 68).

Other types of prior information have been considered for Bayesian image analysis. Gibbs distributions are widely used priors for Bayesian image analysis (38, 78) and also used for Bayesian PET image reconstruction (46, 79). Filipović et al. (46) propose to use the distance-dependent Chinese Restaurant Process (ddCRP) (47) as a prior model in a case of Bayesian PET image reconstruction case, where the full posterior is sampled.

For some choices of prior distribution (where one can evaluate the prior distribution) one can directly compute statistics of the posterior distribution using, for example, least squares inversion, and optimization methods (to find the e.g. MAP model). In these cases, existing optimization based algorithms may by computationally much faster than the proposed sampling algorithm. But for the choices of prior distributions considered for the two cases above, an analytical formulation of the prior does not exist, and hence the prior distribution cannot be evaluated. In these situations optimization methods cannot be readily used.

The proposed method, based on the extended Metropolis sampler, separates the choice of the prior from the sampling algorithm. It does not need a specific mathematical model for the prior and does not need an evaluation of the prior probability. This allows using geostatistical models, GANs, and also e.g. the Gibbs distribution, the ddCRP, and in principle any combination of these to quantify prior information. One can mix and modify the output of any such simulation method, and thus consider a broad set of prior models, to reflect the expectations of a medical expert.

The prior used in the present work is based on the knowledge of a medical expert, thus allowing to consider each realization of the posterior as an example of the actual tracer concentration distribution. This allows medical experts to participate in the construction and evaluation of the specific chosen prior model. The choice of prior is likely the driving factor behind the apparent resolution enhancement reported above. PET standardized uptake values for both normal tissue and in particular cancer can be highly variable, both within and between subjects. For an eventual clinical implementation, prior models should, therefore, be demonstrated to be robust to the choice of parameters of the prior model.

In principle, any collection of independently obtained PET images can be used to represent a prior model. If the sample is large enough it can be used with the proposed methodology, simply by drawing random PET images from the collection in the exploration step of each iteration of the extended Metropolis algorithm. This will then become an example of an independent extended Metropolis sampler, in which one needs only to be able to sample from the prior. Performing a random walk is not necessary. This independent approach is though computationally very inefficient.

One can also use the multiple-point statistical methods to infer conditional higher-order spatial statistics from the collection of realizations (49), or construct a GAN that allows generating realizations with similar spatial statistics (69). In these cases, one can sample the corresponding prior through a random walk, useful in the exploration phase of the proposed algorithm (60, 68).

In the future, we envision a set of generic prior models developed specifically to represent different types of tissue and organs and different diseases. These types of prior models could be distributed to share quantitative prior information.

### 6.2. Perspectives

Detection of small lesions by PET imaging is a well-known and non-trivial diagnostic challenge. The presented methodology can quantify the probability of locating small, high-activity lesions, potentially allowing the identification of cancer lesions at an earlier stage. Our approach may also play a role for treatment response monitoring, with the possibility to quantify not only the activity concentration and lesion size but also the statistical uncertainty of those, which may be useful when analyzing the time evolution of PET images during treatment. This potentially allows quantification of the probability that a lesion has changed in size or activity concentration over time.

The results presented above show the potential of the method, using two examples of prior information. Still, before any preactical clinical application, the method needs to be evaluated on a larger set of data, including Monte Carlo simulation of clinical scenarios.

### 6.3. Current limitations

#### 6.3.1. Stationary and position-invariant convolution operator

In the examples above the convolution operator, G, is assumed to be stationary and position-invariant. And while (12) notes that in many situations the use of a position-invariant point spread function is reasonable, it is an approximation, and ideally, a spatially varying convolution operator should be used (15, 21, 80). Such information can be incorporated in our method by associating each pixel in a PET image by a specific convolution kernel as given by each row in G. The local width of the convolutional operator could be estimated by scanning a known phantom at several locations in the scanner. Further, in the presented methodology, the choice of forward model and noise are closely linked, so limitations of the forward model leading to modeling errors can in principle be taken into account through the likelihood model (81), though such errors may be difficult to quantify.

#### 6.3.2. The noise model

The method requires that a representative noise model can be selected. The PET images considered above were obtained using the 3D OP-OSEM algorithm. Other reconstruction methods exist, such as filtered back projection, MLEM, or recent reconstruction methods based on machine learning such as DeepPET (82), that all lead to reconstructed PET images with different noise properties. While in principle all types of PET images can be used with the proposed methodology, it may be non-trivial to represent the associated noise model for a given choice of linear smoothing operator. Future work will reveal the difficulty related to quantifying such other noise models related to using other PET reconstruction algorithms.

We make use of a multivariate log-normal model to describe the noise, where the noise level is related to the signal level, as also advocated by (32, 52, 83). Figure 3A suggests a slight variability in the magnitude of the noise level from the center to the edge of the PET image that we do not currently model. That should be considered in future work. We estimate the noise variance in two homogeneous areas. Such analysis could be done for more activity levels to get a better model of the distribution of the noise. Here we analyze the noise from a single reconstructed PET image of a known phantom model. Ideally, one could perform multiple PET scans of the same phantom, to get multiple realizations of the noise.

Different PET centers will use different scanners and different reconstruction methods. A known phantom should be scanned regularly for each type of equipment and reconstruction method used, to obtain an optimal noise model and convolution operator. In case this is not possible, a generic noise model could potentially be analytically estimated based on the work of (32, 52).

#### 6.3.3. Computational demands

Sampling methods are known to be computational demanding. This is also the case for the methodology presented here. Sampling the posterior for 2D data subset A of the in vivo case, see Figure 2, takes several hours. On the other hand, sampling the posterior of data subset B takes around 15 min. A simple approach, not considered here, to reduce simulation time is to run multiple shorter independent Markov chains in parallel as opposed to running one single Markov chain as here, utilizing parallel computing capabilities that are constantly being improved.

#### 6.3.4. Extension to 3D

Only 2D cases are considered above. Extension to full 3D is conceptually simple, but in practice, the Monte Carlo based approach will be computationally harder in 3D.^1^ One can though readily analyze, in parallel, a set of 2D slices of data as demonstrated above, from which a pseudo-3D enhanced PET image volume can be obtained.

### 6.4. Probabilistic PET reconstruction using photon count data

Above we have described the PET restoration problem in a probabilistic setting, where the goal is to estimate the true activity concentration distribution from a noisy PET image. The methodology can be used to solve the PET reconstruction problem, where the goal is to estimate the true activity concentration distribution from a set of measured coincidence counts. This will in principle be simple and requires using 1) another forward model, and 2) another noise model, while the prior model would be the same. All corrections normally applied during reconstruction will have to be included in the forward model, but the general solution to this forward problem of simulation of a set of counts from Φ is well-known (84), and the noise model for the (measured) coincidence counts is known to follow a Poisson distribution (5). However, the computational complexity of solving the forward problem will be much higher than when considering the use of a local convolutional operator for PET restoration as we do here. Future research should pursue the ability to perform probabilistic PET restoration, using raw photon count data.

## 7. Conclusion

A probabilistic approach for analysis of the tracer activity concentration from a PET image has been proposed and demonstrated in two examples of phantom and in vivo data. It is based on the conjunction of available information, such as a convolution operator (linked to the point spread function), noise, and an explicit choice of prior information. It has been demonstrated how the convolution operator and noise can be inferred from scanning a known object. Prior information is described by a medical expert and quantified through an algorithm that can simulate examples of tracer uptake reflecting medical expert prior information.

The presented approach allows quantifying the posterior probability of any feature (size, volume, connectivity, activity) simply by analyzing the generated set of images (realizations) of the posterior probability distribution. As an example it has been demonstrated how an image of the average activity concentration can be constructed, that has better resolution and less noise than the original PET image. Also, it has been demonstrated how estimates of both the size and activity of a high-activity lesion can be quantified, as well as the average tracer uptake of a lesion, including estimates of the associated uncertainty.

The proposed methodology allows a quantitative, and probabilistic, approach to PET image analysis, that has the potential to allow a medical expert to identify smaller cancer lesions than possible from the original PET image. Further, the methodology has the potential of being used to monitor the evolution of cancer lesions, as it allows a probabilistic assessment of both the area and activity of cancer lesions, which are important metrics for analyzing the effect of cancer treatment.

## Data Availability

The data analyzed in this study is subject to the following licenses/restrictions: The data are owned by Rigshospitalet, Denmark. Requests to access these datasets should be directed to Adam Espe Hansen, adam.espe.hansen@regionh.dk, Department of Clinical Physiology, Nuclear Medicine and PET, Rigshospitalet, University of Copenhagen, Denmark.

## Footnotes

^1^See supplementary material for an application of the method in 3D to a local 3D subset of phantom data.
