# Supplementary material for: Probabilistic deconvolution of PET images using informed priors
Source: Front Nucl Med. 2023 Jan 12;2:1028928. doi: 10.3389/fnume.2022.1028928 (PMC11459987; doi:10.3389/fnume.2022.1028928)
Supplement: Supplementary file 1 [file Datasheet1.pdf]

Supplementary Material

1 SUPPLEMENTARY DATA

1.1 PET reconstruction using

Figure A1 shows a PET image obatined using a similar PET reconstruction method as in Figure 2, Hansen et al. (2023), but here obtained using a PSF and with a 2 mm Gaussian post-reconstruction filter.

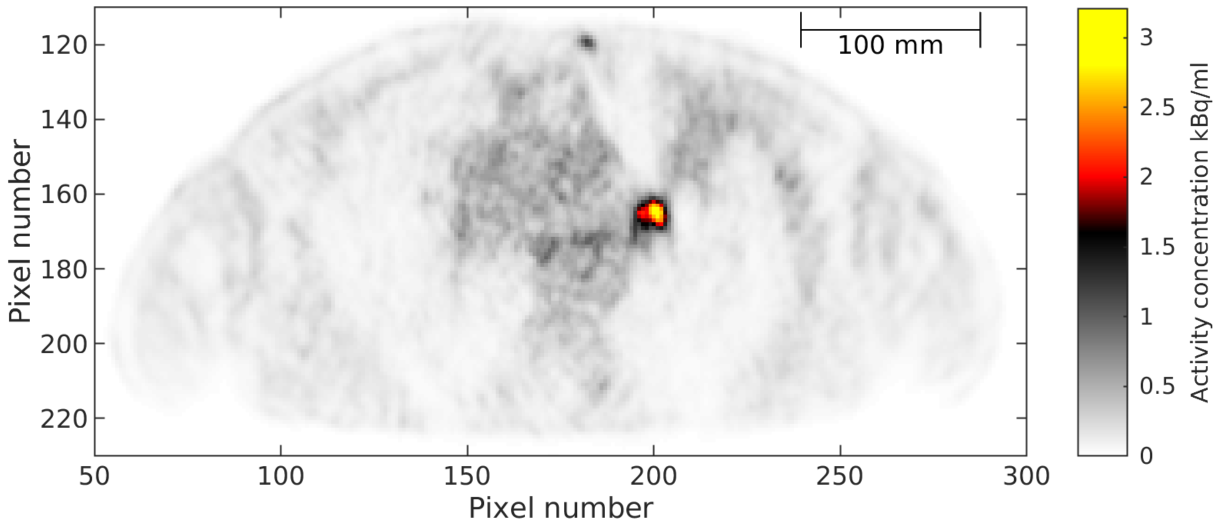

Figure A1. in vivo data. PET image, $\Phi^{\mathrm{obs}}_{\mathrm{PET}_{\mathrm{PSF}}}$ obtained using PSF and with a 2 mm Gaussian post-reconstruction filter. Pixel size is 2.08 x 2.08 mm.

1.2 Comparison to deconvolution

Deconvolution is widely used to infer information from a noisy image that is expected to be the outcome of applying a convolution operator on the original image plus some noise Lavielle (1991); Tohka and Reilhac (2008); Naqa et al. (2006).

Eqn. 3 in the main manuscript describes the relationship between the reconstructed PET image and the in-situ activity as a linear convolution of the in-situ activity plus some noise.

If the noise on the reconstructed PET image as assumed Gaussian $N(0, \mathbf{C}_t)$ and the a prior distribution is also Gaussian $N(\mathbf{\Phi}_0, \mathbf{C}_\Phi)$, the posterior distribution, representing the solution to the deconvolution problem, is also multivariate normal distribution $N(\mathbf{\Phi}_{est}, C_{\mathbf{\Phi}_{est}})$, which can be computed using linear least squares (see e.g. Tarantola and Valette (1982))

$$\mathbf{\Phi}_{est} = \mathbf{\Phi}_0 + \mathbf{C}_\Phi \, \mathbf{G}^T_{PSF} \big(\mathbf{G}_{PSF} \, \mathbf{C}_\Phi \, \mathbf{G}^T_{PSF} + \mathbf{C}_t \big)^{-1} \, (\mathbf{\Phi}_{PET} - \mathbf{G}_{PSF} \, \mathbf{\Phi}_0) \tag{S1}$$

$$C_{\mathbf{\Phi}_{est}} = \mathbf{C}_\Phi \; - \; \mathbf{C}_\Phi \, \mathbf{G}^T_{PSF} \big(\mathbf{G}_{PSF} \, \mathbf{C}_\Phi \, \mathbf{G}^T_{PSF} + \mathbf{C}_t \big)^{-1} \tag{S2}$$

As discussed in the main manuscript the noise is assumed to the multivariate Gaussian in the log-Φ space. Therefore we also assume the multivariate normal prior on Φ to be defined in log-Φ, and assume a constant prior mean and variance as computed from the 1d marginal distribution of $\rho_2(\mathbf{\Phi})$. The covariance structure (i.e. the range of 10 pixels) is the same here. Also, we assumed that $\mathbf{G}_{PSF}$ is a reasonable linear forward operator, also in the log-ϕ space.

This allows using Eqns S1-S2 for directly compute the mean and covariance of the multivariate normal distribution that is the solution to the probabilistic deconvolution problem. Then 100 realizations from this posterior distribution (in log-Φ space) are generated using Cholesky decomposition of $C_{\mathbf{\Phi}_{est}}$, and converted into Φ space. The pointwise mean and standard deviation of the sample of the posterior obtained using deconvolution is compared to the same statistics of $\sigma_1(\phi)$ and $\sigma_2(\phi)$ as described in the main manuscript.

Figure A2 compares the posterior mean (b)-d) and the posterior standard deviation (e)-f) obtained from deconvolution, and $\sigma_1(\Phi)$, and d) $\sigma_2(\Phi)$.

The posterior mean model obtained using deconvolution, Figure A2b, shows less high frequent noise (noise with a longer wavelength), but with a similar amplitude as in the $\mathbf{\Phi}_{PET}$. Using the most informative prior model, $\rho_1(\mathbf{\Phi})$, the posterior man model shows an almost perfect reduction of the noise. Using the less informed prior model leads to a reduction in the amplitude of the noise and a reduction of the high-frequency noise.

It is also apparent that considering $\sigma_1(\Phi)$, and d) $\sigma_2(\Phi)$, most uncertainty is related to the boundary position of the spheres (with only little uncertainty at the centre of the spheres). This is related to the non-Gaussian assumptions about the 1D marginal distribution. Using deconvolution most uncertainty is associated with the pixels with high mean activity (typically at the centre of the spheres) as expected using a 1D Gaussian marginal distribution.

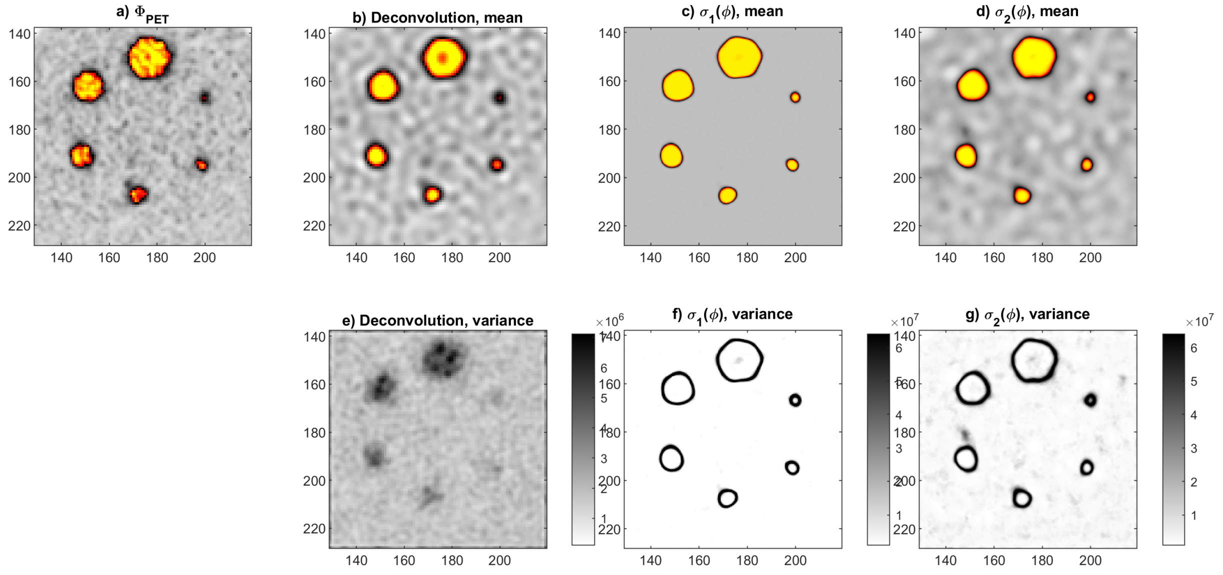

Figure A2. Comparison between a) the reference reconstructed PET image $\mathbf{\Phi}_{PET}$, and the mean of the posterior distribution of activity obtained using b) Bayesian linear deconvolution, c) $\sigma_1(\Phi)$, and d) $\sigma_2(\Phi)$, and the variance of the posterior distribution obtained using e) Bayesian linear deconvolution, f) $\sigma_1(\Phi)$, and g) $\sigma_2(\Phi)$.

1.3 Statistics from $\sigma_2(\Phi)$

Figure 8 in Hansen et al. (2023) show detailed statistics from $\sigma_1(\mathbf{\Phi})$. Figure A3 shows the same types of staistics, but obtained from $\sigma_2(\mathbf{\Phi})$. The results are quite similar except for a a sligtly less pronounced boundary using $\sigma_2(\mathbf{\Phi})$. In addition, the point wise mean activity (column) show some variability stemming from the use of a priori non-constant high and low activity regions.

1.4 Extension to 3D probabilistic PET image analysis

Hansen et al. (2023) propose a probabilistic approach to the analysis of reconstructed PET images, and demonstrate the method on 2D PET images. Extension to 3D is, in principle, straightforward. To illustrate the applicability in 3D, the methodology is demonstrated on smaller 3D subsets of the full 3D reconstructed PET data for the phantom case. As an example prior model 2, $\rho_2(\mathbf{\Phi})$, from Hansen et al. (2023) is considered as the prior model.

Delso et al. (2011) analyze the resolution of the Siemens Biograph mMR scanner used to obtain the data. They conclude that close to the center of the scanner the resolution (in form of the FWHM) is the same in all directions, whereas 10 cm from the center (in the z-direction), the FWHM is about 30% larger in the z-direction than in the x-y direction. As the phantom was placed at the center of the scanner (with some uncertainty) we have chosen to assume that the FWHM of the convolutional operator $\mathbf{G}$ in the z-direction, is the same as in the x-y direction inferred for the 2D case. Ideally, one should analyze a 3D reconstructed PET image relative to a reference model, as done for the 2D case, to obtain the 3D noise model and convolutional operator. Here we use same correlated noise model as for the 2D case, with the same isotropic correlation length in the z-direction as in the x-y-direction

Two 3D data subsets are considered around subset S_6 (the smallest sphere with a 10 mm inner diameter) and subset S_3 (the sphere with a 22 mm inner diameter). See Figure in Hansen et al. (2023) for reference.

Figure A4a shows the 3D PET data used, around subset S_6, with a voxel size of 2.08 x 2.08 x 3.00 mm^3. The voxel size for the model parameters is 1.04 x 1.04 x 1.50 mm^3. Figure A4b shows the 3D point-wise mean of a sample from the posterior distribution. Figure A5 shows point-wise statistics 3D posterior distribution, for the same 2D slice (slice number 84) that was considered for 2D inversion above. Figure A5 can therefore be compared directly to Figure 8f in Hansen et al. (2023).

Figures A6-A7 show similar statistics obtained around subset S_3. Figure A7 can be compared to the results obtained using 2D PET data in Figure 8c in Hansen et al. (2023)

These preliminary results suggest that using the full 3D PET reconstructed image provides better resolution, less entropy, than using only 2D reconstructed PET image. The point-wise mean and probability of locating high intensity is more pronounced in Figure A5 than in Figure 8f in Hansen et al. (2023). This is not surprising, due to the expected correlation in the vertical direction of both the noise and the point-spread function. However, any such conclusions can only be made when a more detailed analysis of the 3D point-spread function and noise model is done. We leave this for future research.

REFERENCES

Delso, G., Fürst, S., Jakoby, B., Ladebeck, R., Ganter, C., Nekolla, S. G., et al. (2011). Performance measurements of the siemens mmr integrated whole-body pet/mr scanner. *Journal of nuclear medicine* 52, 1914–1922

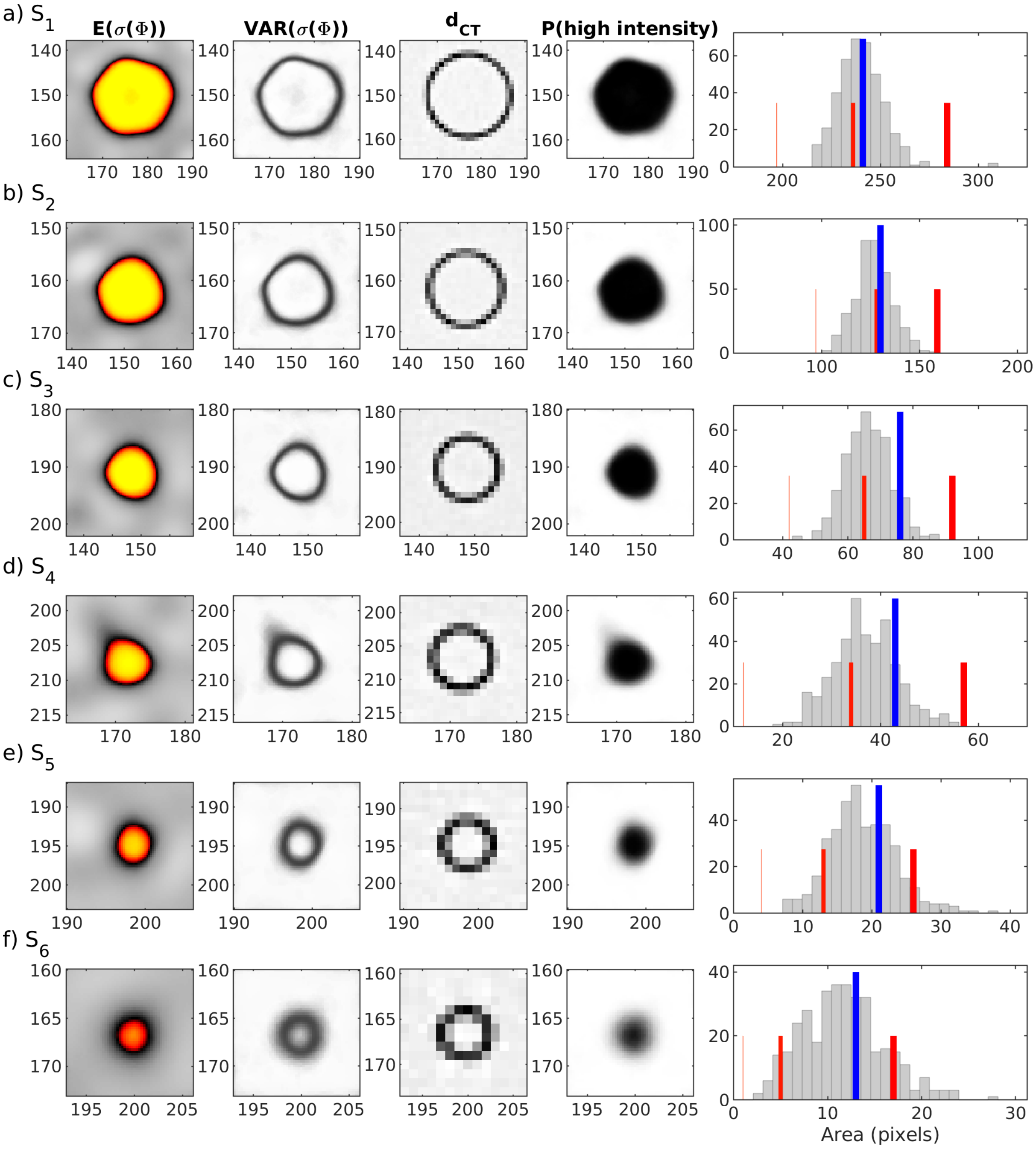

Figure A3. Statistics from $\sigma_2(\mathbf{\Phi})$, around the 6 spheres (S_1-S_6). Column 1: Pointwise mean activity. Column 2: Pointwise variance (black:high, white:low). Column 3: CT data. Column 4: Posterior probability of having high activity (> 1.5 kBq/ml) (black:1, white:0). Column 5: Posterior probability of the area of the region with high activity (> 1.5 kBq/ml). The blue line indicates the area obtained from the CT image. Red lines indicate areas obtained by thresholding the reconstructed PET image at levels > 1.0 (thick), > 1.5 (medium), and > 2.0 kBq/ml (thin). Compare to Figure 8 in Hansen et al. (2023).

Hansen, T. M., Mosegaard, K., Holm, S., Andersen, F., Fischer, B. M., and Hansen, A. E. (2023). Probabilistic deconvolution of PET images using informed priors. *Frontiers in Nuclear Medicine* doi:10.3389/fnume.2022.1028928

Lavielle, M. (1991). 2-D bayesian deconvolution. *Geophysics* 56, 2008–2018

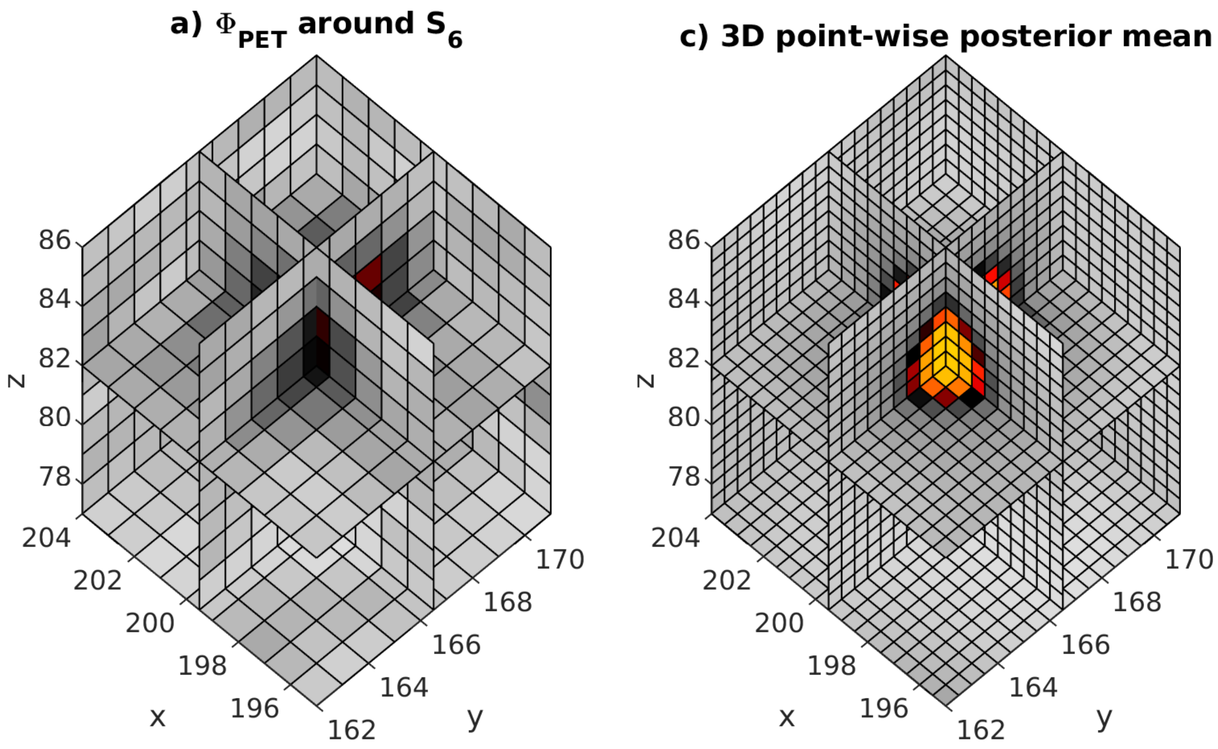

Figure A4. Left) 3D PET data around data subset S_6. 3D Point-wise mean of the posterior distribution $\sigma_2(\mathbf{\Phi})$

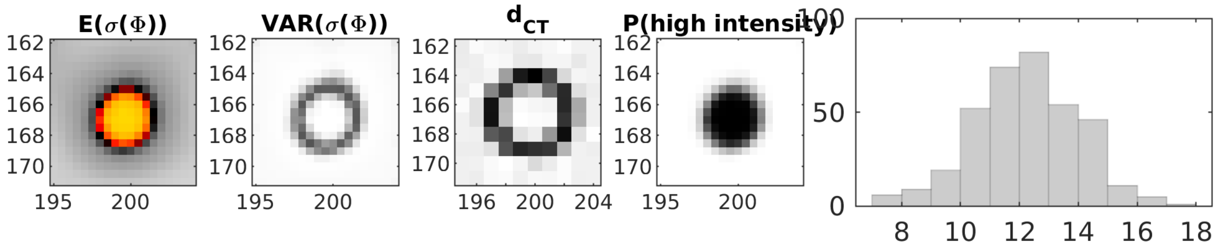

Figure A5. Posterior 2D statistics (of PET slice 82) obtained from 3D inversion around subset S_6. From left to right: Point-wise posterior mean, variance, 2D PET image, point-wise probability of locating high intensity, and a histogram of the area of high intensity. Compare to results obtained in 2D in Figure 8f in Hansen et al. (2023).

Naqa, I. E., Low, D. A., Bradley, J. D., Vicic, M., and Deasy, J. O. (2006). Deblurring of breathing motion artifacts in thoracic PET images by deconvolution methods. *Medical physics* 33, 3587–3600

Tarantola, A. and Valette, B. (1982). Generalized nonlinear inverse problems solved using the least squares criterion. *Reviews of Geophysics* 20, 219–232

Tohka, J. and Reilhac, A. (2008). Deconvolution-based partial volume correction in Raclopride-PET and Monte Carlo comparison to MR-based method. *Neuroimage* 39, 1570–1584

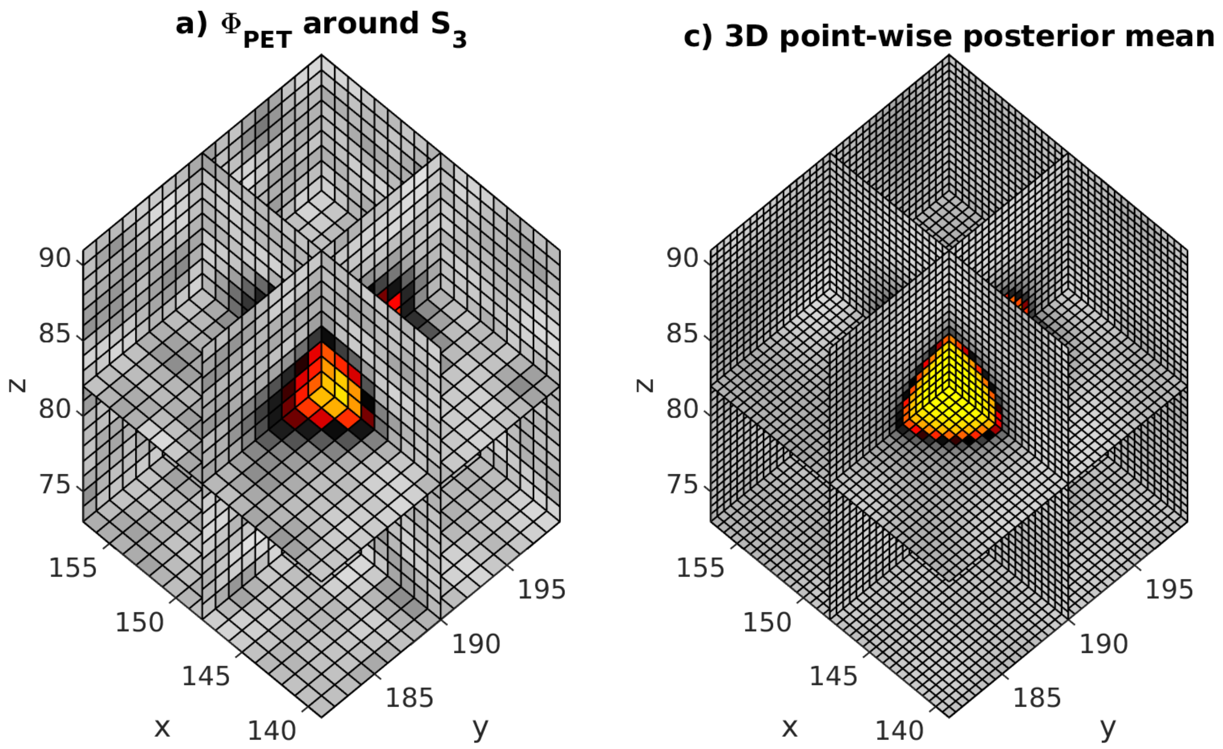

Figure A6. Left) 3D PET data around data subset S_3. 3D Point-wise mean of the posterior distribution $\sigma_2(\mathbf{\Phi})$

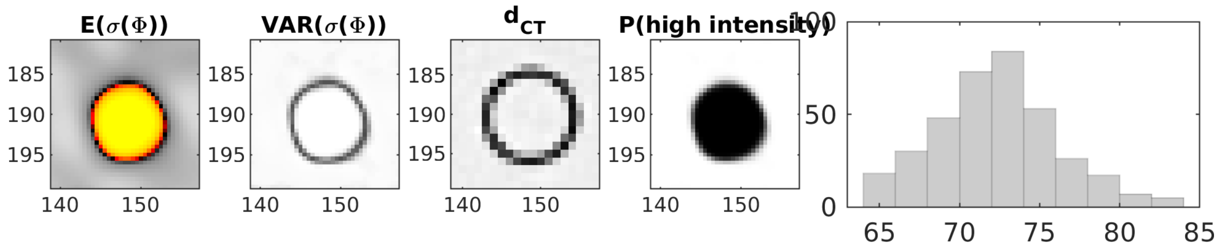

Figure A7. Posterior 2D statistics (of PET slice 82) obtained from 3D inversion around subset S_3. From left to right: Point-wise posterior mean, variance, 2D PET image, point-wise probability of locating high intensity, and a histogram of the area of high intensity. Compare to results obtained in 2D in Figure 8c in Hansen et al. (2023).
